# The Conformations of Isolated Gallic Acid: A Laser-Ablation Rotational Study

**DOI:** 10.3390/molecules28010159

**Published:** 2022-12-24

**Authors:** Andrés Verde, Susana Blanco, Juan Carlos López

**Affiliations:** Departamento de Química Física y Química Inorgánica, Facultad de Ciencias, IU CINQUIMA, Universidad de Valladolid, 47011 Valladolid, Spain

**Keywords:** polyphenols, phenolic acids, bioactive molecules, antioxidant molecules, rotational spectroscopy, laser ablation

## Abstract

The rotational spectrum of laser-ablated gallic acid has been recorded using CP-FTMW spectroscopy. Two rotamers have been detected, and their rotational spectra have been assigned and analyzed to obtain the molecular spectroscopic parameters. The observed rotamers have been unambiguously identified in the light of theoretical computations, based on the comparison of the experimental line intensities and rotational parameters with the rotational constants and electric dipole moments predicted from theoretical calculations. The values of the planar inertial moments confirm that the observed conformers are planar, and their relative stability and population have been determined from relative intensity measurements. The B3LYP-D3/6-311++G(2d,p) level has been shown to be the best method among a series of levels normally used to predict the rotational parameters in rotational spectroscopy. In the observed conformers, the three adjacent OH groups are arranged in a sequential form, and the only difference between them lies in the orientation of the COOH group. Although weak attractive OH···O interactions seem to exist, the analysis of the electron density topology does not show the existence of any critical point corresponding to these interactions.

## 1. Introduction

Natural polyphenols are secondary metabolites found in most parts of plants with functions in growth and development, and mechanisms against pathogens or UV rays. They are also responsible for color, acidity, odor, or taste [1]. Their biological potential and health-promoting properties has been attributed to their antioxidant and metal chelation capacity, and to the direct modulation of cell signaling pathways [2,3,4]. Polyphenols possess at least one aromatic ring and one or more hydroxyl groups, including derivatives such as methoxy groups and glycosides. These can be classified according to the number of phenol rings and the way these rings bind each other. A simple classification divides polyphenols into flavonoids and non-flavonoids, and the latter into phenolic acids, stilbenes and lignans [5]. 

Antioxidants are substances that prevent, inhibit, or delay the oxidation of other substances. In biological systems, antioxidants contribute to removing free radicals and reducing oxidative stress. Antioxidant molecules may follow different defense mechanisms. The antioxidant activity of polyphenols depends on their structure and on the type and position of substituents [6,7]. Polyphenols with substituents such as OH, O–R, COOH, and C=O may have chelating activity, so they may chelate ions such as Fe^+2^ or Cu^+^ which prevent the reactions leading to the formation of hydroxyl radical, one of the most dangerous reactive oxygen species [8]. Furthermore, as polyphenols have multiple OH groups, they are involved in mechanisms of scavenging free radicals either by donating a hydrogen free radical (H^.^) or donating an electron (e^−^) [8]. The resonance and hyperconjugation effects affecting the π orbitals of the aromatic ring or CO groups, and the lone pairs of oxygen atoms contribute to stabilizing the transient secondary free radicals. This stability is essential for a compound to be an effective antioxidant [9,10].

**Scheme 1:**
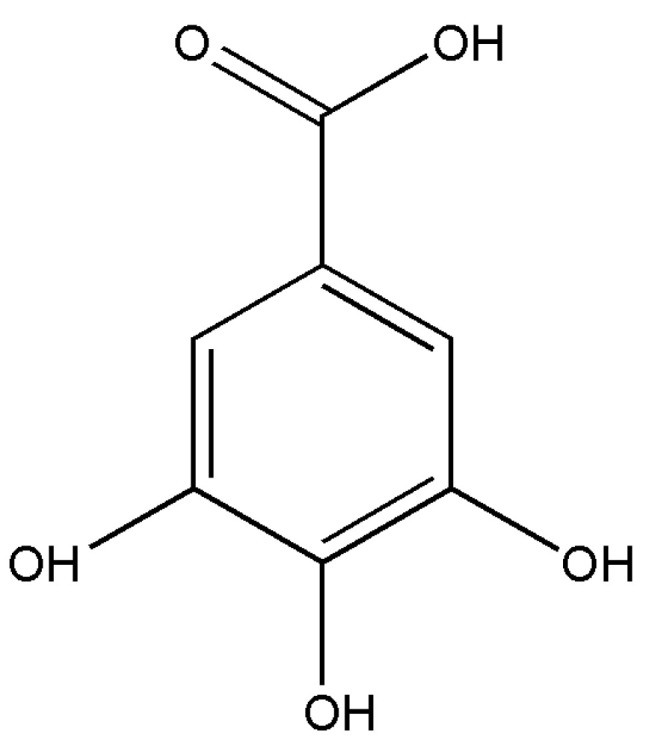
Gallic acid.

Gallic acid (GA, 3,4,5-trihydroxy benzoic acid, see Scheme 1) is a phenolic acid that owes its name to its first isolation from oak galls [11]. It is found in nearly every part of plants, free and as a component of tannins. GA is used in tanning, ink dyes, paper manufacturing, or enology, but due to its antioxidant properties [12,13,14,15] it has attracted interest in the field of pharmacology in the treatment of different diseases [16,17,18,19,20,21,22,23]. The GA molecule has a phenyl ring with three hydroxyl groups *ortho* to each other, and a carboxylic group structure that gives GA a strong antioxidant capacity [24]. The structure of GA and its monohydrate has been the object of X-Ray studies [25,26,27,28,29,30,31,32,33,34] in crystal forms and complexed to enzymes [35,36]. There are several theoretical studies on GA that have investigated its structure, anionic and hydrated forms, and antioxidant properties [37,38,39,40,41,42,43,44]. These theoretical studies conclude that there are four possible conformations of GA in isolation, but there is no experimental information on this molecule in the gas phase, where the molecules are free of the intermolecular interactions established in condensed phases.

Accurate structural information of molecules in the gas phase can be obtained using rotational spectroscopy. This includes a variety of techniques that record the spectra associated with transitions between rotational states, which occur in the microwave (MW) region. The rotational constants, and thus the moments of inertia of the rotating molecules, can be directly obtained from the analysis of the rotational spectra. These parameters depend on the coordinates and masses of the nuclei forming the molecule. Rotational spectroscopies make possible the unambiguous discrimination between different isomers, those being tautomers, conformers, or isotopomers. The spatial mass distribution of any of those species has individual spectroscopic constants and unique individual rotational spectra, a feature that makes rotational spectroscopy a very powerful tool. In the past, the application of this technique was mainly limited to stable molecular specimens of moderate size, with a permanent dipole moment and possessing appreciable vapor pressures. However, the development of Fourier transform microwave (FTMW) spectroscopies, combined with supersonic jets, has contributed to overcoming many of these limitations with reinforced high resolution and sensitivity [45,46,47]. Supersonic jets provide the collision-free environment conditions needed to analyze the intrinsic structural properties of a molecule. Molecules expanding in the supersonic jet cool down the vibrational degrees of freedom to populate the lowest energy vibrational state. In this context, molecules that have several minima in their potential energy surface (PES) corresponding to different stable conformers, experiment this cooling process within each of the PES wells. This allows the different conformers to be effectively isolated and tested with different spectroscopic techniques. Challenging molecular systems have been studied, including a variety of weakly bound molecular complexes, unstable molecular species generated in situ, or solid biomolecules generated by using electrical discharge [48] or laser ablation [49], respectively. The rotational spectra of very low dipole moment systems are now accessible, and the detection of molecules with zero dipole moment is possible if the molecule can form adducts with polar ligands such as water [50]. Thus, at present, FTMW spectroscopies can be considered one of the most definitive gas-phase structural probes. 

In this work, we present a study of the rotational spectrum of GA using chirped-pulse Fourier transform spectroscopy. The main drawbacks to bringing GA into the gas phase are the low vapor pressure, high melting (258 °C), and boiling (501 °C) points. Although GA sublimates at 210 °C, it decomposes into pyrogallol and CO_2_. We have used laser ablation [51,52] to overcome these problems to vaporize GA.

## 2. Results and Discussion

### 2.1. Conformational Search

**Figure 1:**
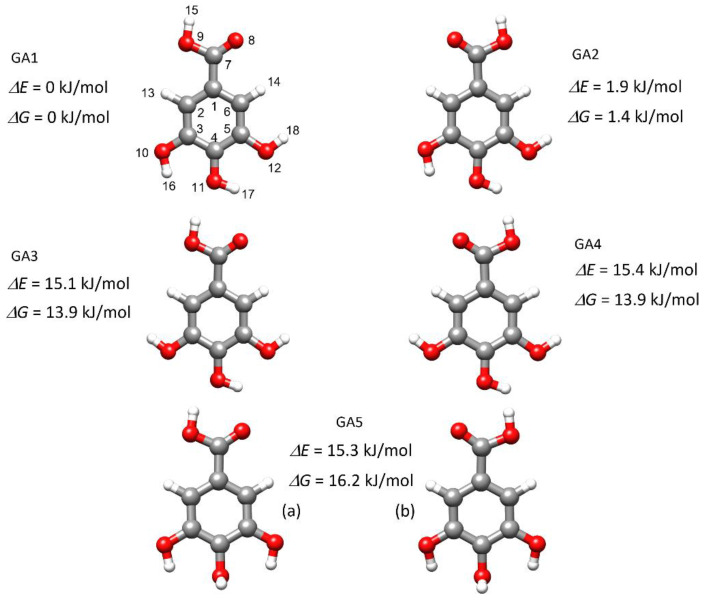
The five stable conformers predicted for GA together with the relative electronic and Gibbs free energies calculated at the B3LYP-D3/6-311++G(2d,p) level. For conformer GA5, the two equivalent atropisomers (a) and (b) are shown.

Before analyzing the rotational spectrum of GA, some theoretical computations were done to predict the parameters needed to calculate the rotational spectrum. B3LYP and MP2 methods with different basis sets were benchmarked with the experimental data. The five stable conformers found for GA are shown in Figure 1. The corresponding spectroscopic parameters are given in Tables S1–S6. Four of them, GA1 to GA4, were reported in previous works [41]. GA1 and GA2 forms are predicted to have nearly the same energy and the same rotational parameters, independently of the method used. In both cases, the three adjacent hydroxyl groups are in the ring plane and present the same orientation in a sequential arrangement. The only difference between them is the orientation of the carboxyl group. The potential energy profile for the rotation of the COOH group connecting these forms is depicted in Supplementary Materials: Figure S1. The arrangement of the hydroxyl groups in conformers GA3 and GA4, with a different orientation of the OH groups in position 3, leads to a higher energy than forms GA1 and GA2. The potential energy profiles for the OH group rotation connecting forms GA1–GA3 and GA2–GA4 are shown in Figure S2. In form GA5, with predicted energy close to those of forms GA3 and GA4, the central OH group in position 4 is perpendicular to the ring, and the lateral OH groups in positions 3 and 5 point toward the central oxygen atom. The rotation of the COOH group by 180° gives rise to two equivalent atropisomers (a) and (b) (see Supplementary Materials: Figure S3). In the literature on X-Ray diffraction structures, GA1 [26,30] and GA2 [28,29,30,31,33,34] are found both for GA and its monohydrate, with GA2 being the most frequently reported form. GA3 [28,30,32] and GA4 [28,32] are reported generally for the monohydrate. To our knowledge, no report on form GA5 exists, although it could be related to the mechanism in which the O-H bond in position 4 is homolitically broken [39] by the reaction of GA with a radical.

### 2.2. Rotational Spectrum 

**Figure 2:**
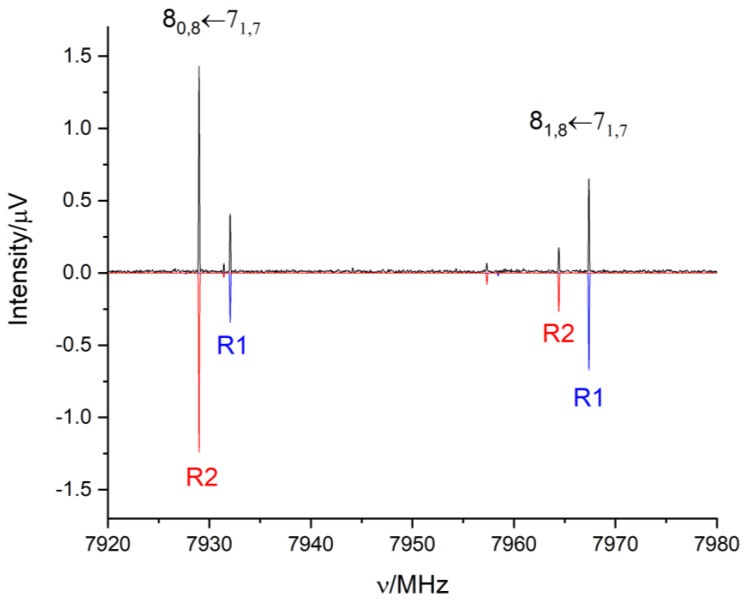
A section of the CP-FTMW spectrum of laser ablated GA showing the 8_1,8_ ← 7_1,7_ (*a*-type) and 8_0,8_ ← 7_1,7_ (*b*-type) transitions. The experimental spectrum (upper black trace) is compared with the spectrum predicted with the fitted rotational parameters (see Table 1) for rotamers R1 (lower blue trace) and R2 (lower red trace).

The jet-cooled rotational spectrum of laser-ablated GA is shown in Figure S4. The spectrum lines were easily assigned from the predictions of the spectrum, which was done using the theoretical rotational constants for the most stable conformers of GA (see Figures S5 and S6). These are predicted to be prolate asymmetric rotors with values of the Ray asymmetry parameter [53] of *κ* = −0.66 and non-zero *μ*_a_ and *μ*_b_ electric dipole moment components. As shown in Figure 2, the spectrum is composed of couples of lines close in frequency, but with different intensities corresponding to two rotamers labeled R1 and R2, with similar rotational constants. Both rotamers have *a*- and *b*-type transitions. The initial assignment was done using the JB95 program [54], while the final measurement of the spectrum was done using the AABS (Assignment and Analysis of Broadband Spectra) package written by Kisiel [55,56], and the SPFIT/SPCAT program suite written by H. M. Pickett [57] for the fitting and prediction of rotational spectra. Both detected spectra were analyzed with the S-reduced Watson’s semirigid Hamiltonian in the I^r^ representation [58]. The resulting parameters are given in Table 1. The measured frequencies are given in Tables S12 and S13. When dropping the lines measured from the spectrum, it becomes practically clean with no lines attributable to other possible forms of GA or derived fragments.

## 3. Discussion

The determined rotational parameters are quite close to the theoretically calculated values for the predicted conformers (see Table 1 and Tables S1–S6), which only differ in the position of the hydrogen atoms. Although the predicted energies let us think that the observed rotamers are conformers GA1 and GA2, a comparison of the experimental and theoretical rotational constants is not the best way to identify the conformers. Fortunately, the orientation of the electric dipole moment in the principal inertial axis system, and thus the *μ*_a_, *μ*_b_, and *μ*_c_ components, are strongly dependent on the orientation of the COOH or the OH groups. The low values of the electric dipole moment components for GA3 form, the low value of *μ*_a_ for GA4, and the non-zero *μ*_c_ component for GA5 definitively allow us to discard these three high-energy forms. An analysis of the electric dipole moment component values for GA1 and GA2, and the observed relative intensities observed for rotamers R1 and R2, allow for a definitive identification of rotamer R1 as conformer GA1 and rotamer R2 to form GA2. The values of the *μ*_a_ electric dipole moment are predicted to be nearly equal for conformers GA1 and GA2, so the line strengths for the *a*-type rotational transitions would also be nearly equal for both observed rotamers. In the spectrum, the lines of the *a*-type lines of rotamer R1 are more intense than those of rotamer R2, as shown in Figure 2 for the 8_1,8_ ← 7_1,7_ transitions. We have analyzed the relative intensities between rotamers, considering that the observed line intensities are proportional to the square of the electric dipole moment component driving the selection rules, and to the population of the corresponding conformers in the supersonic expansion.

Relative intensity (*I*) measurements for the *a*-type transitions give an average value of *I*_GA2_/*I*_GA1_ = 0.29 ± 0.02. Using the B3LYP-D3/6-311++G(2d,p) *μ*_a_ values, the relative populations (*N*) of the conformers is *N*_GA2_/*N*_GA1_ = 0.30 ± 0.02. The average of the relative intensity measurements for the b-type lines is *I*_GA2_/*I*_GA1_ = 2.5 ± 0.2. Using the relative populations calculated from the a-type lines a ratio *μ*_b_(GA2)/*μ*_b_(GA1) = 2.9 ± 0.2 is calculated, not far from the B3LYP-D3/6-311++G(2d,p) predicted ratio of 4.2 D/1.4 D = 2.96. The population ratio calculated from the *b*-type lines relative intensities and the predicted dipole moments is *N*_GA2_/*N*_GA1_ = 0.29±0.02, consistent with that calculated from the a-type lines. The average population for this ratio calculated from both *a*- and *b*-type spectra is *N*_GA2_/*N*_GA1_ = 0.30±0.02. The observed relative abundance of conformers GA1 and GA2 in the supersonic expansion is the result of a series of processes that include the laser vaporization, the seeding of molecules in the region where the laser ablation plume and the carrier gas stream cross each other, and the collisional cooling occurring in the subsequent supersonic expansion. Laser ablation is a complex process that may result in high temperatures and subsequent cooling. The relative population of the different conformers of gallic acid would be brought close to that of thermodynamic equilibrium at the temperature of the carrier gas, only if a high collision rate exists in the seeding region [59]. Using the B3LYP-D3/6-311++g(2d,p) vibrational and rotational data, we have analyzed the temperature and pressure dependence of the equilibrium relative population ratio. While this ratio has a small dependence on pressure, it is sensitive to temperature changes, as seen in Figure S7. The ratio observed in the supersonic jet corresponds to temperatures around 155 K. We do not know the amount of each form in the solid sample used, but if a high collisional rate exists in the seeding region at ca. 7.5 mm from the 0.8 mm nozzle, the observation of a population ratio corresponding to equilibrium at a temperature lower than the stagnation temperature can be reasonable [60]. The intensities of the observed lines are evidence to support the identification of the observed rotamers R1 and R2 as GA1 and GA2, respectively. 

The GA1 and GA2 conformers are predicted to be planar at equilibrium. The predicted planar moment of inertia *P*_c_ = (*I*_a_ + *I*_b_ − *I*_c_)/2 = Σ_i_*m*_i_c_i_^2^ should be zero for a planar molecule. The experimental values of the planar moment *P*_c_ have values of 0.27570(18) uÅ^2^ for GA1 and 0.28558(20) uÅ^2^ for GA2. These values correspond to the ground vibrational state of each conformer. They have values different from zero due to out-of-plane vibrational contributions, as those that can arise from vibrations associated to the COOH and OH groups are bonded to the ring. In benzoic acid [61], which is also planar, the experimental value of *P*_c_ is 0.1829(1) uÅ^2^, and in catechol [62] with two adjacent OH groups the value is 0.04716(2) uÅ^2^. Comparison of these values with those for the observed conformers of GA let us conclude that both conformers are planar. The planarity of the observed forms is very important concerning the antioxidant properties of GA [15,41], since it maximizes resonance and hyperconjugation effects between the aromatic ring or C=O group π orbitals and the oxygen atom lone pair orbitals. Going back to the observed rotational constants, it could be observed that the method that better predicts their values is B3LYP-D3/6-311++G(2d,p), followed by MP2 with the same basis set. The values predicted with this DFT method are compared to the experimental values in Table 1. Based on this agreement, the B3LYP-D3/6-311++G(2d, p) structures can be taken as good descriptions of the geometries of GA1 and GA2 conformers. The DFT geometries for all five GA1-GA5 conformers are given in Tables S7–S11.

The nature of the potential energy function associated with the interconversion between the different GA forms has its primary origin in the resonance stabilization effects, which are maximized for arrangements of the COOH and OH groups coplanar with the benzene ring. The barriers appearing for the different functions shown in Figures S1–S3 correspond to perpendicular arrangements either of the COOH or the OH groups. The energy differences between forms GA1 and GA2 can be attributed to the different orientation of the COOH group relative to the sequential arrangement of the O-H groups. A possible contribution to stabilize conformer GA1 may come from the electrostatic interaction between the dipoles of the COOH and OH groups. This can be deduced from the values of the *μ*_b_ dipole moment component, since both forms have nearly the same value of *μ*_a_. The total dipole generated by the OH groups is practically directed along the *b-axis*. In GA1 form the *μ*_b_ component is smaller than in GA2, indicating that for the latter the *μ*_b_ component of the COOH group dipole has the same sign as the OH group dipole component. For GA1 both components have a contrary sign, giving an extra stabilization to this conformer.

**Figure 3:**
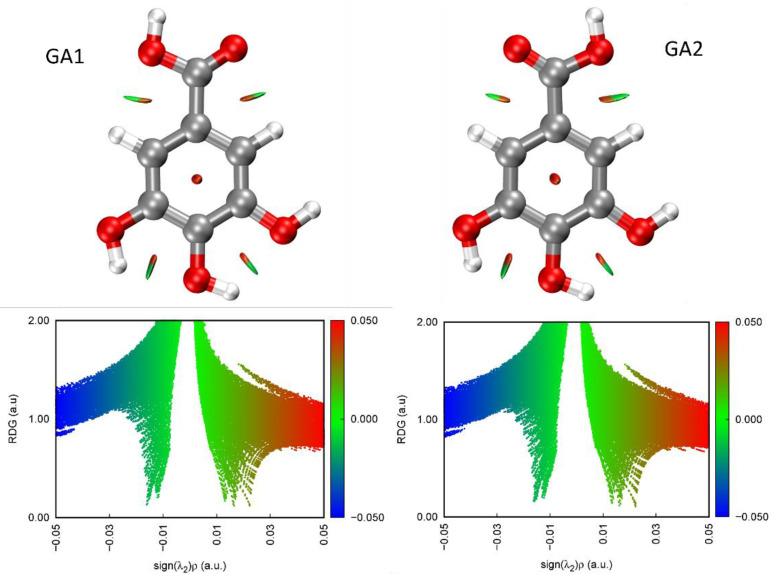
The figure shows the results of the non-covalent interaction (NCI) analysis applied to conformers GA1 and GA2. The equilibrium structures calculated at the B3LYP-D3/6-311++G(2d,p) has been considered.

To gain information on possible non-covalent interactions (NCI) contributing stabilize the forms of GA, NCI [63,64], analyses were done for the different calculated conformers using the B3LYP-D3/6-311++G(2d,p) level. The results for conformers GA1 and GA2 are shown in Figure 3. Those for the rest of the conformers are given in Figures S8 and S9. Each point in the scatter graphs is a grid point in 3D space, representing the reduced density gradient (RDG) *vs*. sign[*λ*_2_]*ρ*. *λ*_2_ is the largest second eigenvalue of the Hessian matrix of the electron density *ρ*. The strength of weak interactions has a positive correlation with the electron density in the corresponding region. Van der Waals interaction regions always have very small values of *ρ*, while the regions corresponding to strong steric effects and hydrogen bonding always have relatively large values of *ρ*. A negative sign of *λ*_2_ indicates attractive interactions since electron density is aggregated. A positive sign of *λ*_2_ indicates repulsive interactions in which the electron density depletes. Thus, the product of the sign of *λ*_2_ and *ρ* allows visualizing the non-covalent interactions. The spikes in the low part of the scatter graphs represent the non-covalent interactions present. Those on the left part of the graph correspond to attractive interactions, and those on the right part correspond to repulsive interactions. Points with RGD < 0.5 a.u., are represented in the isosurfaces, and the strength of the interaction is identified with the colour codes shown in the scatter graphs.

Weak O-H ···O attractive contacts coexisting with O ···O repulsive interactions between the OH groups in the sequential arrangement are evident in all cases (see Figure 3, Figure S8 and Figure S9). These attractive interactions practically disappear in conformers GA3 and GA4 (see Figure S8) for the OH groups showing different orientations, probably contributing to destabilizing these forms. Weak C-H···O attractive and C···O repulsive interactions are observed to occur between the carboxyl group and the adjacent C-H groups in the benzene ring. 

**Figure 4:**
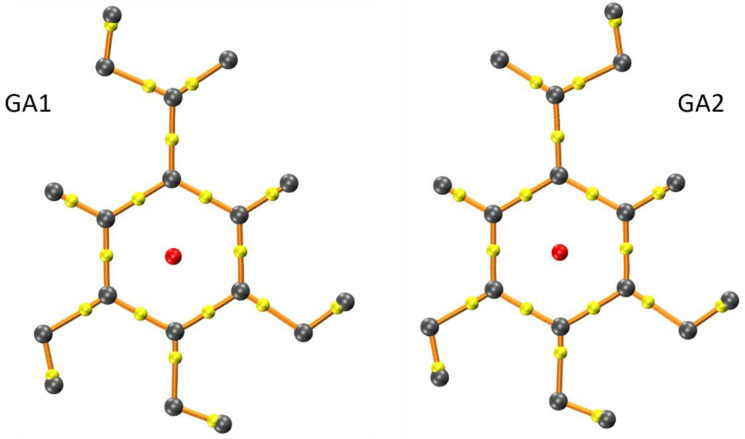
Molecular graph showing the results of the QTAIM analysis of conformers GA1 and GA2 of GA. Critical points are depicted with different colors, grey for the (3,−3) maxima locating the atoms, yellow for the (3,−1) bond critical points (BCP), and red for the (3,1) ring critical points (RCP). Bond paths (BP) are shown in orange. The equilibrium structures calculated at the B3LYP-D3/6-311++G(2d,p) has been considered. As can be seen, there are no BCP locating possible hydrogen bonds between OH groups.

Bader’s quantum theory of atoms in molecules (QTAIM) [65] analyses reveal the structure through the stationary points of the electron density function *ρ*(*r*), together with the electron density gradient paths connecting these points. The localization of maxima allows the identification of atomic positions, whereas saddle points between the maxima, called bond critical points (BCP), define chemical bonds. QTAIM analysis is one of the theoretical methods proposed to reveal the existence of hydrogen bond interactions from the corresponding BCP [66]. Some pieces of evidence of the existence of weak hydrogen bond interactions between the hydroxyl group of GA were found in a previous work from NBO computations [41]. However, as can be seen in Figure 4 and Figure S10, QTAIM analysis does not reveal the existence of any bond critical points different from those associated with conventional covalent bonds of the molecule. This indicates that apparently there are no hydrogen bonds connecting the OH groups in GA if we take the existence of BCP as fingerprints of these bonds. In GA1 form, the O···H distances are predicted to be r(O_12_···H_17_) = 2.205 Å and r(O_11_···H_16_) = 2.207 Å and the O···O distances r(O_11_···O_12_) = 2.706 Å and r(O_10_···O_11_) = 2.721 Å. The angles O-H···O are ∠O_11_H_17_O_12_ = 111° and ∠O_10_H_16_O_11_ =112°. These distances and angles correspond to hydrogen bonds in the limit between moderate and weak interactions [66]. However, due to hyperconjugation effects stabilizing this geometry, the planar configuration of the hydroxyl groups does not correspond to the expected hydrogen bond geometry maximizing the strength of the interaction along the axis of one of the oxygen lone pairs as conventionally envisaged; that is with a geometry closer to a tetrahedral arrangement around the acceptor oxygen atom. In any case, the NCI analysis indicates the existence of weak attractive interactions which, to some extent, could be considered as weak hydrogen bonds. The existence of these interactions is evident from the fact that when one of these disappears, as occurs in conformers GA3 and GA4 (see Figure S8), the molecule becomes more unstable than when it adopts forms GA1 and GA2.

## 4. Materials and Methods

### 4.1. Experimental

The first experimental steps to record the GA rotational spectrum in the 2–8 GHz frequency range were performed using a chirped pulse Fourier transform microwave spectrometer (CP-FTMW) with a heatable nozzle. Although the sample was heated to temperatures above 180 °C, it did not vaporize and therefore its spectrum did not appear. This fact is explained by its physical properties (solid, mp 250 °C, bp 500 °C). In addition, GA decomposes upon heating into pyrogallol and CO_2_ [38]. Faced with these problems, the laser ablation vaporization method was used as a better alternative to seed GA molecules into the supersonic jet stream [51,52].

In the laser ablation experiment, a solid rod was prepared by grinding a 1:1 mixture of the pure GA sample-Cu powder, and then pressed to form cylindrical rods of approximately 9 mm in diameter. This rod was held in a special laser ablation nozzle, which consists of a standard solenoid valve with the nozzle coupled to a homemade extension cap. The light pulses from a Q-switch Nd/YAG laser (Quantel Q-smart 850, 5.2 ns pulse width) were focused on the rod through a small orifice of the nozzle. The laser was used in its second harmonic, with pulse energies of approximately 20 mJ per pulse. In order to ensure that the laser hit a different point of the sample surface during the experiment, the rod was continuously translated and rotated by a stepper motor. When the sample was vaporized, it was dragged by the carrier gas (Ar, 5 bar stagnation pressure) expanding into the high vacuum chamber as a supersonic jet due by the pressure gradient. Once in the vacuum chamber, the molecules were excited using a 4 μs long shaped frequency chirp generated by an arbitrary waveform generator, emitted by a horn antenna and amplified by a TWT amplifier in the 2 to 8 GHz range. After that, the molecules spontaneously emitted a free induction decay (FID) signal that was detected through another horn, recorded with a digital oscilloscope and Fourier transformed from the time domain to the frequency domain. The polarization-detection sequence was repeated up to eight times per molecular expansion, obtaining eight averaged spectra in every molecular jet pulse. The repetition rate of molecular pulses was 5 Hz, allowing optimal vacuum conditions in the chamber. The spectrum recorded is the result of the accumulation of 1 million spectra.

### 4.2. Theoretical

The conformational landscape of GA was explored using the DFT and MP2 methods. The B3LYP hybrid functional of Becke [67], Lee, Yang, and Parr [68] with 6-311++G(2d,p) [69] and def2-TZVP [70] basis sets and including GD3 [71] and GD3BJ [72] empirical dispersion corrections. Furthermore, *ab initio* MP2 [73] optimizations were done with 6-311++G(2d,p), aug-cc-pVDZ, and aug-cc-pVTZ [74] basis sets. Harmonic approximation calculations were also carried out to determine the vibrational frequencies and confirm that all the conformers were indeed minima. Relaxed scan calculations were done at the B3LYP-GD3/6-311++(2d,p) level to explore the internal rotation coordinates through which interconversion between the stable conformers occurs. All these calculations were done using Gaussian 16 package [75].

In order to analyze the nature of all the intramolecular interactions that stabilize the structures, the quantum theory of atoms in molecules (QTAIM) [65] and non-covalent interaction (NCI) [64] analyses were done using Multiwfn program [63] with the B3LYP-GD3/6-311++G(2d,p) results.

## 5. Conclusions

We have studied the rotational spectrum of GA using CP-FTMW spectroscopy, which is based on the use of supersonic jets. GA is solid, has very low vapor pressure and high melting and boiling points. In addition, it decomposes into pyrogallol and CO_2_ upon heating following a pyrolysis reaction [38]. To avoid these problems, we have succeeded by seeding the sample molecules into the supersonic expansion by using laser ablation. Two rotamers have been detected, and their rotational spectra have been assigned and analyzed to obtain the molecular spectroscopic parameters. The unambiguous identification of the observed rotamers as GA1 and GA2 conformers has been based on the comparison of the experimental spectral features such as rotational constants, planar moments of inertia, and observed intensities, with the rotational constants and electric dipole moments predicted from theoretical calculations. The values of the planar inertial moments show that these conformers are planar, as was theoretically predicted. The planarity of the observed forms is very important concerning the antioxidant properties of GA. A planar molecule maximizes resonance and hyperconjugation effects between the aromatic ring or C=O group π orbitals and the oxygen atom lone pair orbitals. In free radical scavenging reactions, these resonance effects allow the delocalization of the secondary free radical electron, thus increasing stability [9,10]. The conformers interconvert through the internal rotation of the acid group, with a barrier high enough to preclude any collisional relaxation process in the supersonic jet. The stability of GA1 over GA2 has been shown experimentally from relative intensity measurements, which allow the determination of the relative conformer populations in the supersonic jet. B3LYP-D3/6-311++G(2d,p) level has been shown to be the best method to predict the rotational parameters among a series of levels conventionally used to predict the rotational parameters in rotational spectroscopy. On the basis of the theoretical predictions, we have discussed the existence of hydrogen bond interactions between the OH groups of GA. Although weak OH···O interactions seem to exist, QTAIM analysis of the topology of the electron density of GA does not show the existence of any critical point corresponding to these interactions.

## Figures and Tables

**Table 1:** Rotational parameters obtained from the analysis of the spectrum of the two conformers of GA and its comparison with the DFT values (B3LYP-GD3/6-311++G(2d,p)) for the identified forms.

Param. ^a^	Rotamer R1	GA1	Rotamer R2	GA2
*A*/MHz	1602.84558(20) ^b^	1602.4	1602.69867(25)	1602.3
*B*/MHz	663.46993(10)	663.6	663.13549(10)	663.3
*C*/MHz	469.477690(86)	469.3	469.30622(10)	469.1
*κ*	−0.66	−0.66	−0.66	−0.66
*P*_a_/uÅ^2^	761.44548(18)	761.6	761.81977(20)	762.0
*P*_b_/uÅ^2^	315.02548(18)	315.4	315.04450(20)	315.4
*P*_c_/uÅ^2^	0.27570(18)	0.0	0.28558(20)	0.0
*D*_J_ /kHz	0.00690(86)	0.0054	0.00605(63)	0.0054
*D*_JK_ /kHz	0.0190(20)	0.0427	0.0274(26)	0.0428
*D*_K_ /kHz	0.037(11)	0.0202	0.039(13)	0.0202
*d*_1_ /kHz	0.00209(23)	−0.0019	0.00253(33)	−0.0019
*d*_2_ /kHz	0.000510(88)	0.0000	0.00025(11)	−0.0005
*σ*/kHz	3.1		3.7	
*n*	133		116	
*μ*_a_/D	moderate	1.9	moderate	1.8
*μ*_b_/D	moderate	1.4	intense	4.2
*μ*_c_/D	no	0.0	no	0.0

^a^ A, B and C are the rotational constants; *κ* is Ray’s asymmetry parameter (−1 < *κ* = (2*B* − *A − C*)/(*A − C*) < 1) [53]; *P*_αα_ (*α* = *a*, *b* or *c*) are the planar moments of inertia derived from the inertial moments; *I*_α_ (*P*_cc_ = (*I*_a_ + *I*_b_ − *I*_c_)/2 ); *D*_J_, *D*_JK_, *D*_K_, *d*_1,_ and *d*_2_ are the quartic centrifugal distortion constants; σ is the rms deviations of the fit; n is the number of lines fitted; *μ*_a_, *μ*_b_, and *μ_c_,* are the components of the electric dipole moment along the principal inertial axes; “moderate”, “intense” or “no” correspond to the intensities observed for the corresponding *a*- *b*- or *c*-type spectra. ^b^ Standard errors are given in parentheses in units of the last digit.

## Data Availability

The data presented in this study are available in this paper and the corresponding Supplementary Material.

## Supplementary Materials

The following supporting information can be downloaded at: https://www.mdpi.com/article/10.3390/molecules28010159/s1, Complete reference [75]; Figures S1–S3 shows the potential energy function for the internal rotation of the COOH group and OH groups interconverting different low-energy forms. Figures S4–S6 show the GA rotational spectrum compared to predicted spectra. Figure S7 shows temperature dependence of the equilibrium relative population of the conformers observed. Figures S8–S10 give the results of NCI and QTAIM analysis GA3–GA5 GA forms. Tables S1–S6 list the spectroscopic parameters and energies predicted for the different conformers using several theoretical levels. Tables S7–S11 contain the *r*_e_ structures for the different forms of GA. Tables S12 and S13 contain the observed frequencies and residuals.
