# Supplementary material for: The Conformations of Isolated Gallic Acid: A Laser-Ablation Rotational Study
Source: Molecules. 2022 Dec 24;28(1):159. doi: 10.3390/molecules28010159 (PMC9822196; doi:10.3390/molecules28010159)
Supplement: Supplementary file 1 [file molecules-28-00159-s001.zip › molecules-2087187-supplementary.pdf]

The conformations of isolated gallic acid: a laser-ablation rotational study

Andrés Verde [1], Susana Blanco [1] and Juan Carlos López [1,*]

[1] Departamento de Química Física y Química Inorgánica, IU CINQUIMA, Facultad de Ciencias, Universidad de Valladolid, 47011 Valladolid, Spain; andres.verde@estudiantes.uva.es, susana.blanco@uva.es, juancarlos.lopeza@uva.es

* Correspondence: juancarlos.lopeza@uva.es; Tel.: +34 983185891

Complete Reference [74]

Figures S1-S3. B3LYP-D3/6-311++G(2d,p) potential energy profiles for the interconversion of conformers GA1-GA2 (S2), GA1-GA3, GA2-GA4 (S3), GA5a-GA5b (S4) of gallic acid.

Figure S4-S6. The CP-FTMW spectrum of laser ablated gallic acid in the 3-8 GHz region. In Figure S4 is compared to the fitted spectra, in Figure S5 to the DFT predicted spectra for conformers GA1-GA5, and in Figure S6 to the sum of DFT predicted spectra with intensities corrected using expected population ratios at room temperature.

Figure S7. Temperature dependence of the equilibrium relative population ratio N_{GA2}/N_{GA1} of conformers GA1 and GA2 calculated from the predicted B3LYP-D3/6-311++G(2d,p) vibrational and rotational data.

Figure S7-S8 Results of the NCI analysis for the predicted GA3, GA4 and GA5 conformers of gallic acid.

Figure S10. Molecular graph showing the results of the QTAIM analysis of conformers GA3, GA4 and GA5 of gallic acid.

Tables S1-S6. Predicted Rotational parameters, electric dipole moment components, and energies for the five conformers of gallic acid, calculated at different levels of theory.

Table S7-S11. r_e geometry for the different conformers of gallic acid calculated at the B3LYP-D3/6-311++G(2d,p) for the five predicted conformers of gallic acid.

Table S12-S13. Observed rotational transitions and residuals (all the values in MHz) for the observed rotamers of gallic acid.

Complete Reference [74]

Gaussian 16, Revision A.03, Frisch, M. J.; Trucks, M. J.; Schlegel, H. B.; Scuseria, G. E.; Robb, M. A.; Cheeseman, J. R.; Scalmani, G.; Barone, V.; Petersson, G. A.; Nakatsuji, H.; Li, X.; Caricato, M.; Marenich, A. V.; Bloino, J.; Janesko, B. G.; Gomperts, R.; Mennucci, B.; Hratchian, H. P.; Ortiz, J. V.; Izmaylov, A. F.; Sonnenberg, J. L.; Williams-Young, D.; Ding, F.; Lipparini, F.; Egidi, F.; Goings, J.; Peng, B.; Petrone, A.; Henderson, T.; Ranasinghe, D.; Zakrzewski, V. G.; Gao, J.; Rega, N.; Zheng, G.; Liang, W.; Hada, M.; Ehara, M.; Toyota, K.; Fukuda, R.; Hasegawa, J.; Ishida, M.; Nakajima, T.; Honda, Y.; Kitao, O.; Nakai, H.; Vreven, T.; Throssell, K.; Montgomery Jr., J. A.; Peralta, J. E.; Ogliaro, F.; Bearpark, M. J.; Heyd, J. J.; Brothers, E. N.; Kudin, K. N.; Staroverov, V. N.; Keith, T. A.; Kobayashi, R.; Normand, J.; Raghavachari, K.; Rendell, A. P.; Burant, J. C.; Iyengar, S. S.; Tomasi, J.; Cossi, M.; Millam, J. M.; Klene, M.; Adamo, C.; Cammi, R.; Ochterski, J. W.; Martin, R. L.; Morokuma, K.; Farkas, O.; Foresman, J. B.; Fox, D. J. Gaussian, Inc., Wallingford CT, 2016.

Figure S1. B3LYP-D3/6-311++G(2d,p) potential energy profile for the rotation of the COOH group of gallic acid interconverting conformers GA1-GA2 (see Figure 1). (1 kJ/mol = 83.5935 cm^{-1}).

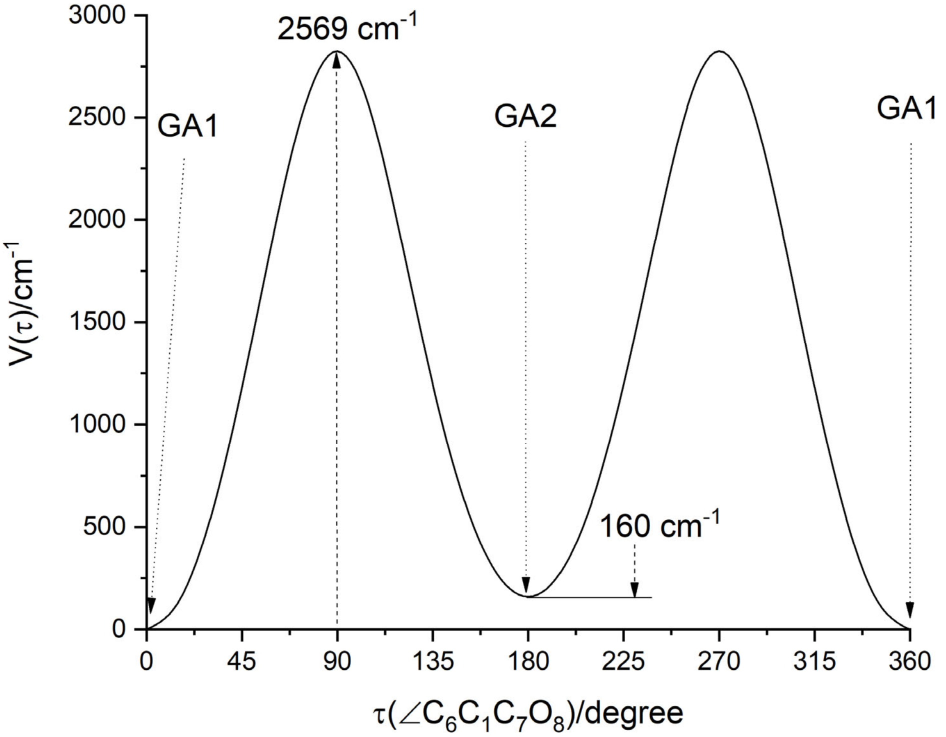

Figure S2. B3LYP-D3/6-311++G(2d,p) potential energy profile for the rotation of the $O_{10}H_{16}$ group of gallic acid interconverting conformers GA1-GA3 as well as GA2-GA4 (see Figure 1). (1 kJ/mol = 83.5935 cm^{-1}).

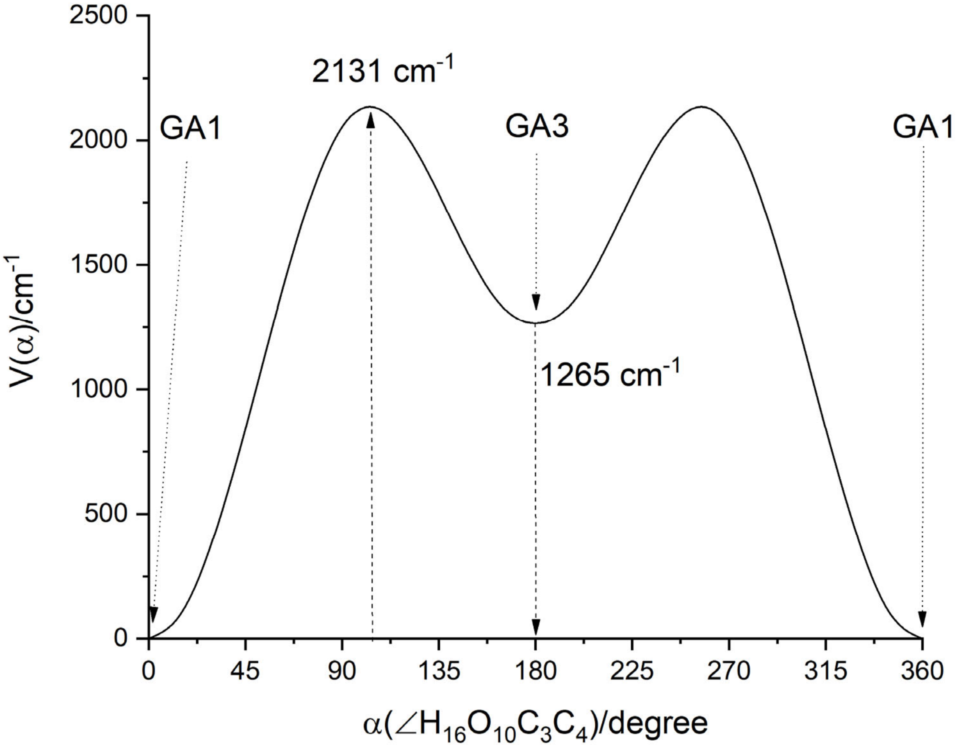

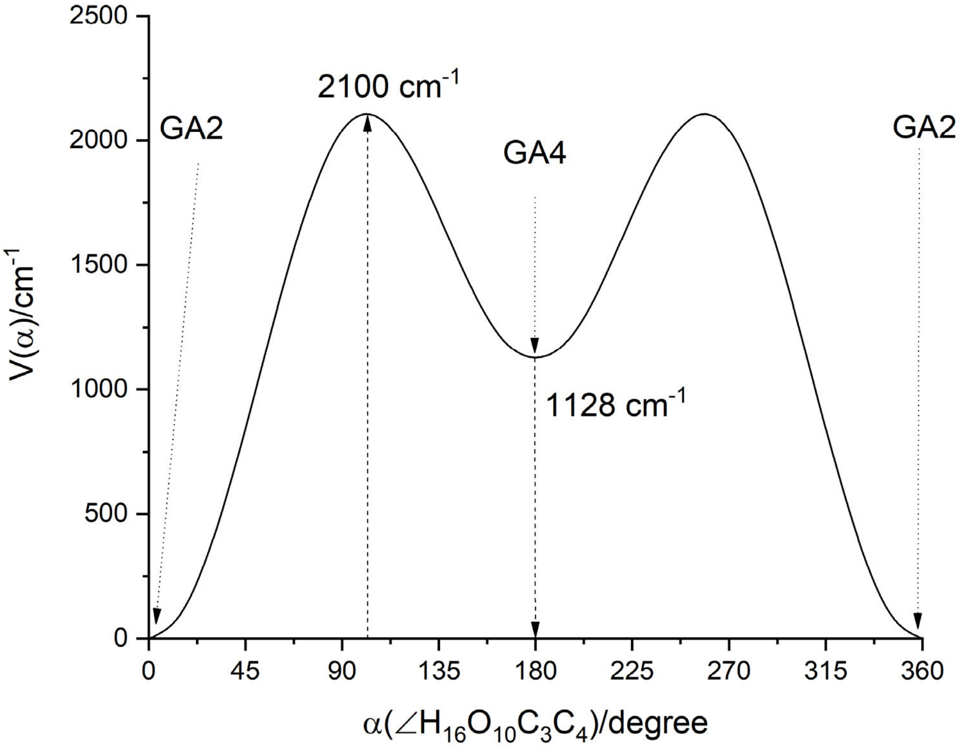

Figure S3. B3LYP-D3/6-311++G(2d,p) potential energy profile for the rotation of the COOH group of gallic acid conformers GA5 interconverting atropisomers (a) and (b) (see Figure 1). (1 kJ/mol = 83.5935 cm^{-1}).

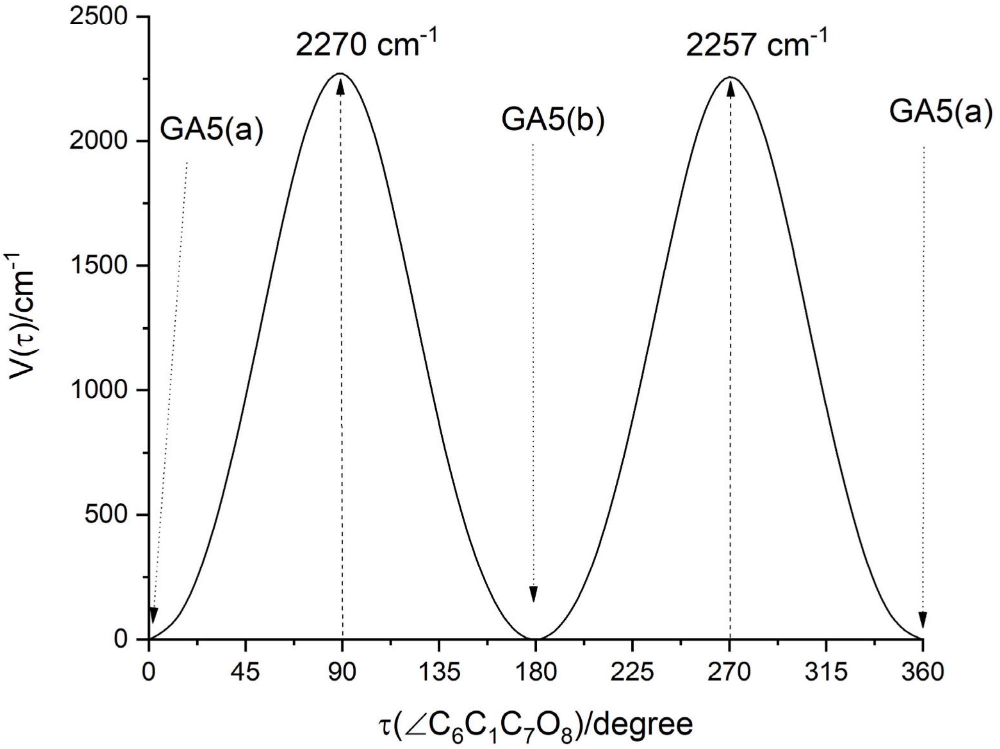

Figure S4. The CP-FTMW spectrum of laser ablated gallic acid in the 3-8 GHz region. The experimental spectrum (upper black trace) is compared with the spectrum predicted with the fitted rotational parameters (see Table 1) for rotamers R1 (lower blue trace) and R2 (lower red trace).

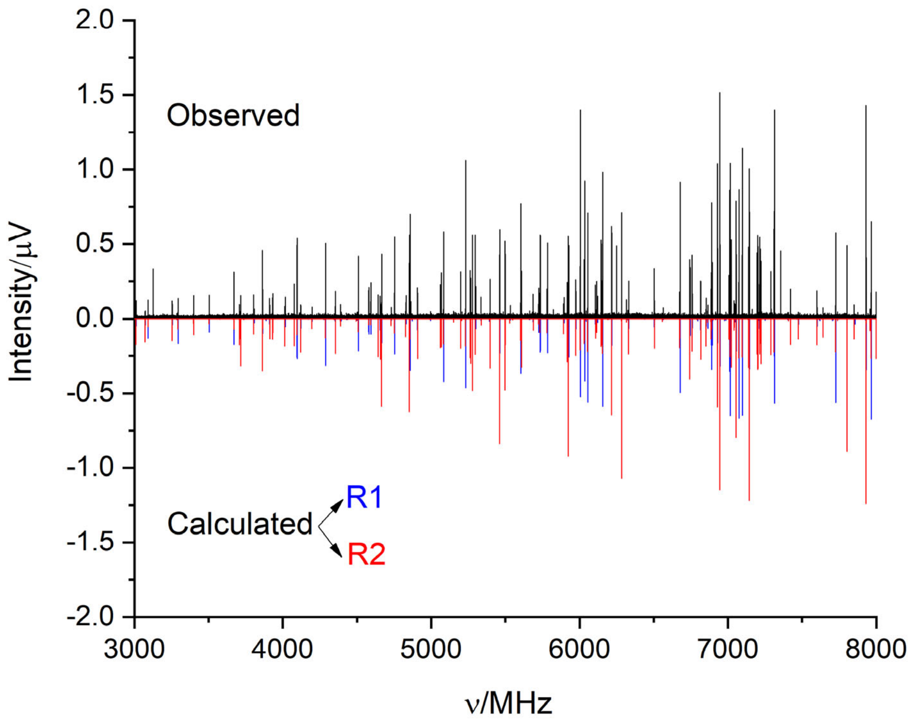

Figure S5. The CP-FTMW spectrum of laser ablated gallic acid in the 3-8 GHz region. The experimental spectrum (upper black trace) is compared with the spectra predicted from the DFT B3LYP-D3/6-311++G(2d,p) rotational parameters (see Table 1) for conformers GA1 (blue trace), GA2 (red trace), GA3 (navy blue trace), GA4 (dark yellow trace) and GA5 (dark gray trace) conformers. The calculated spectra intensities correspond exclusively to the calculated electric dipole components and have not been corrected for estimated populations. The spectrum of GA3 form thus corresponds to dipole moment values of μ_a=0.3 D**,** μ_b=0.0 D, μ_c=0.0 D.

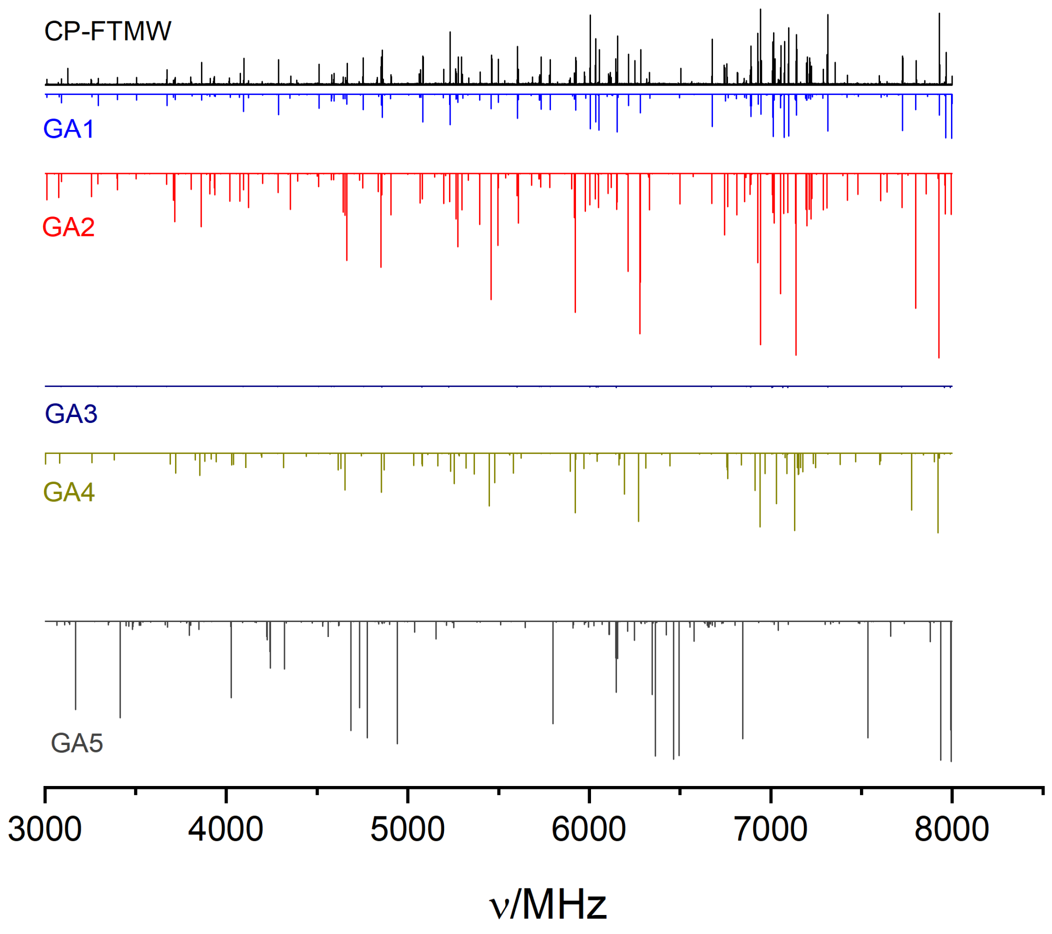

Figure S6. The CP-FTMW spectrum of laser ablated gallic acid in the 3-8 GHz region. The experimental spectrum (upper black trace) is compared with the sum of spectra predicted from the DFT B3LYP-D3/6-311++G(2d,p) rotational parameters (see Table 1) for conformers GA1 (blue trace), GA2 (red trace), GA3 (navy blue trace), GA4 (dark yellow trace) and GA5 (dark gray trace) conformers. The calculated spectra intensities have been corrected using relative populations predicted from Gibbs energies. Only spectra for conformers GA1 and GA2 have appreciable intensities and alone reproduce the observed spectrum.

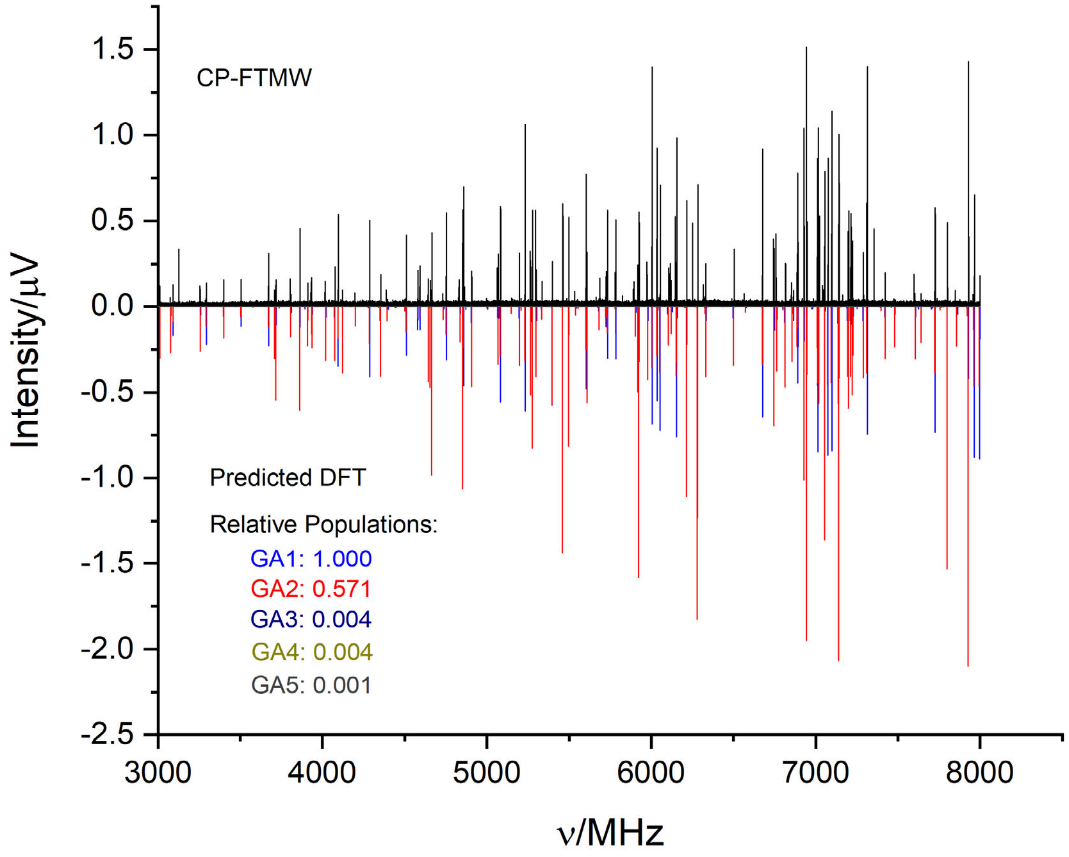

Figure S7. Temperature dependence of the equilibrium relative population ratio N_{GA2}/N_{GA1} of conformers GA1 and GA2 calculated from the predicted B3LYP-D3/6-311++G(2d,p) vibrational and rotational data. According to these predictions the ratio of 0.3 observed in the supersonic expansion would correspond to a temperature of 155 K.

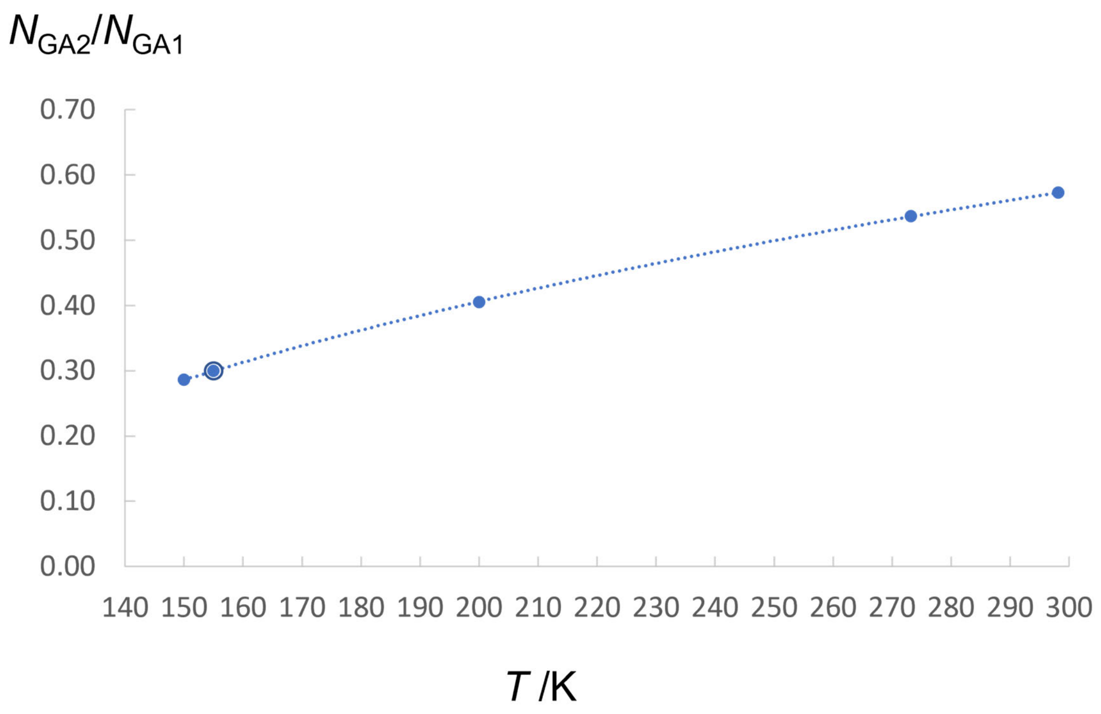

Figure S8. The figure shows the results of the non-covalent interaction (NCI) analysis (E. R. Johnson, *et al.*, *J. Am. Chem. Soc.* **2010**, *132*, 6498–6506) applied to conformers GA3 and GA4 of gallic acid. The equilibrium structure calculated at the B3LYP-D3/6-311++G(2d,p) has been considered. Each point in the scatter graph (see below) is a grid point in 3D space representing the reduced density gradient (RDG) *vs*. sign[λ_2]ρ. λ_2 is the largest second eigenvalue of the Hessian matrix of the electron density ρ. The strength of weak interactions has a positive correlation with the electron density in the corresponding region. Van der Waals interaction regions always have very small ρ values, while the regions corresponding to strong steric effects and hydrogen bonding always have relatively large values ρ. A negative sign of λ_2 indicates attractive interactions since electron density is aggregated. A positive sign of λ_2 indicates repulsive interactions in which the electron density depletes. Thus, the product of the sign of λ_2 and ρ allows visualizing the non-covalent interactions. The spikes in the low part of the scatter graphs represent the non-covalent interactions present. Those on the left part of the graph correspond to attractive interactions and those on the right part correspond to repulsive interactions. Points with RGD < 0.5 a.u., are represented in the isosurface and the strength of the interaction is identified with the color codes shown below.

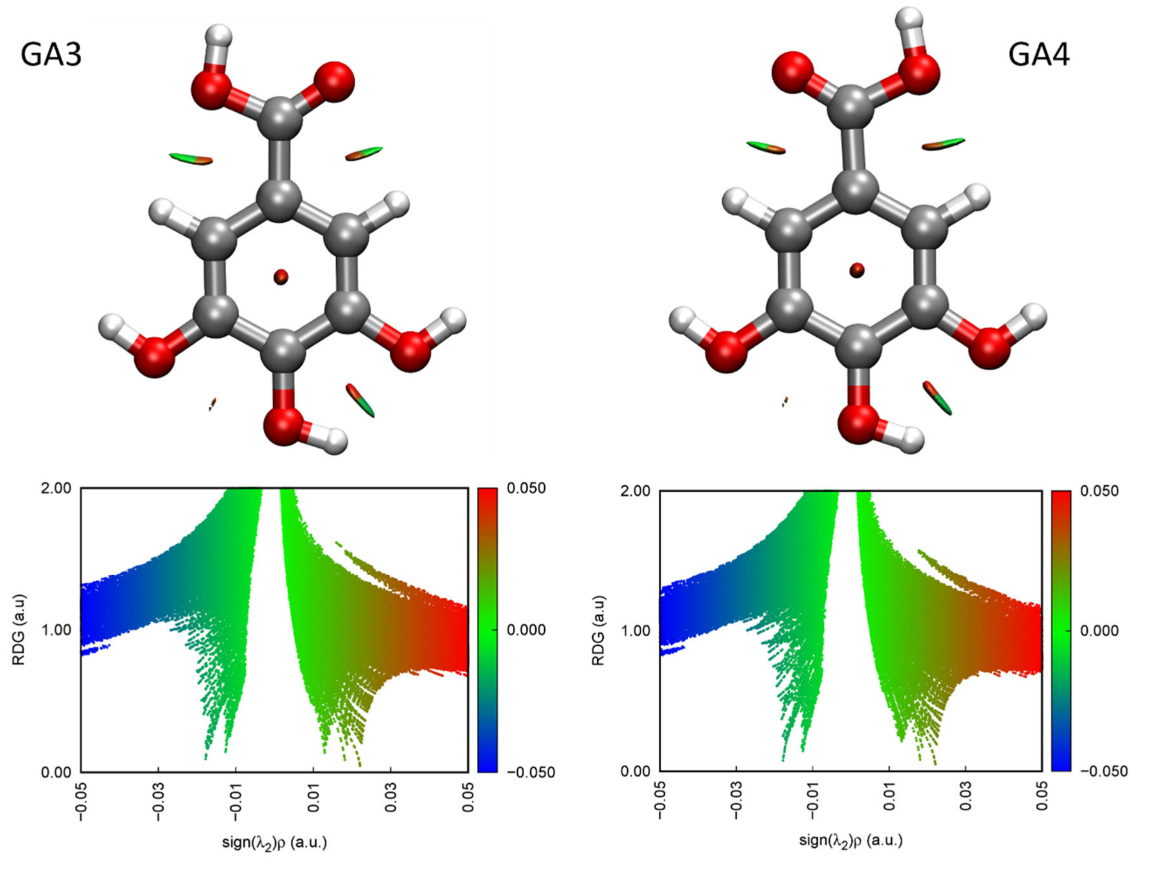

Figure S9. The figure shows the results of the non-covalent interaction (NCI) analysis (E. R. Johnson, *et al.*, *J. Am. Chem. Soc.* **2010**, *132*, 6498–6506) applied to conformers GA5 (*a*) and (*b)* of gallic acid. The equilibrium structure calculated at the B3LYP-D3/6-311++G(2d,p) has been considered. Each point in the scatter graph (see below) is a grid point in 3D space representing the reduced density gradient (RDG) *vs*. sign[λ_2]ρ. λ_2 is the largest second eigenvalue of the Hessian matrix of the electron density ρ. The strength of weak interactions has a positive correlation with the electron density in the corresponding region. Van der Waals interaction regions always have very small ρ values, while the regions corresponding to strong steric effects and hydrogen bonding always have relatively large values of ρ. A negative sign of λ_2 indicates attractive interactions since electron density is aggregated. A positive sign of λ_2 indicates repulsive interactions in which the electron density depletes. Thus, the product of the sign of λ_2 and ρ allows visualizing the non-covalent interactions. The spikes in the low part of the scatter graphs represent the non-covalent interactions present. Those on the left part of the graph correspond to attractive interactions and those on the right part correspond to repulsive interactions. Points with RGD $<$ 0.5 a.u., are represented in the isosurface and the strength of the interaction is identified with the color codes shown below.

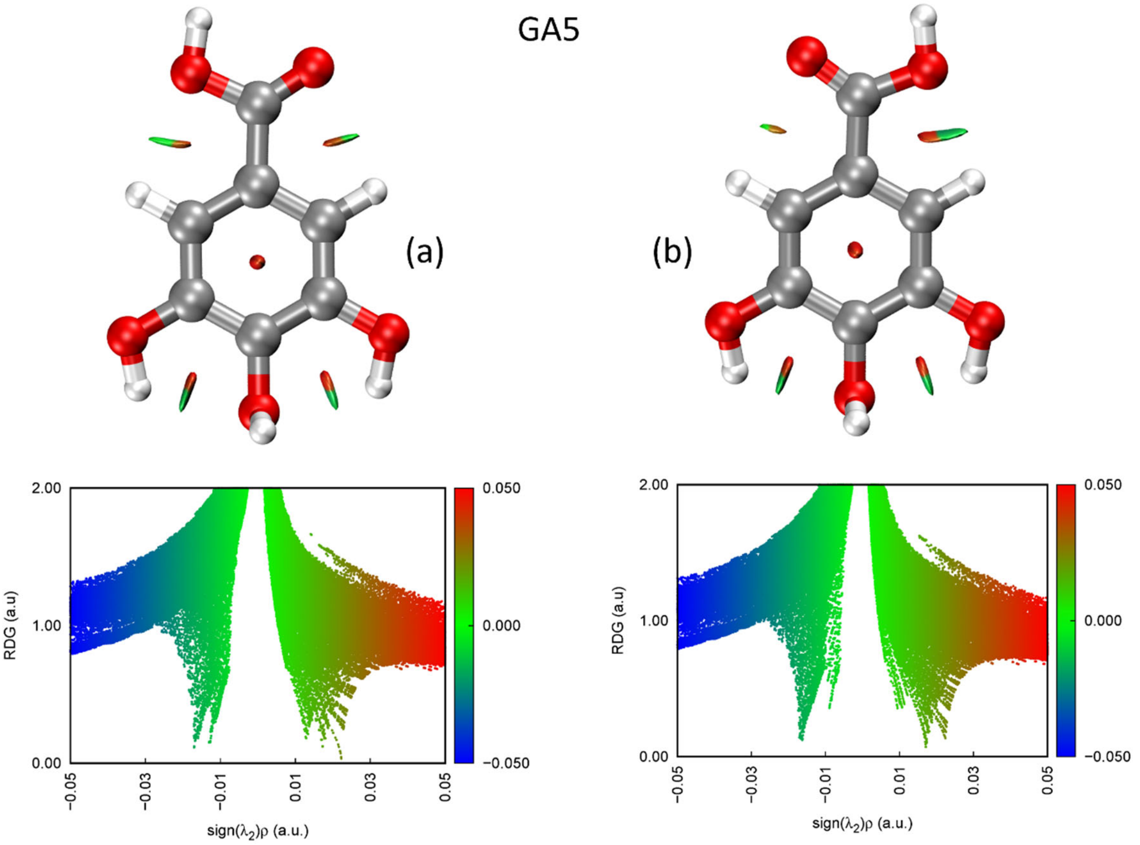

Figure S10. Molecular graph showing the results of the QTAIM analysis of conformers GA3, GA4 and GA5 of gallic acid. Critical points are depicted with different colors, grey for the (3,-3) maxima locating the atoms, yellow for the (3,-1) bond critical points (BCP), and red (3,1) ring critical points (RCP). Bond paths (BP) are shown in orange. The equilibrium structures calculated at the B3LYP-D3/6-311++G(2d,p) has been considered. As can be seen, there are no BCP locating possible hydrogen bonds between OH groups.

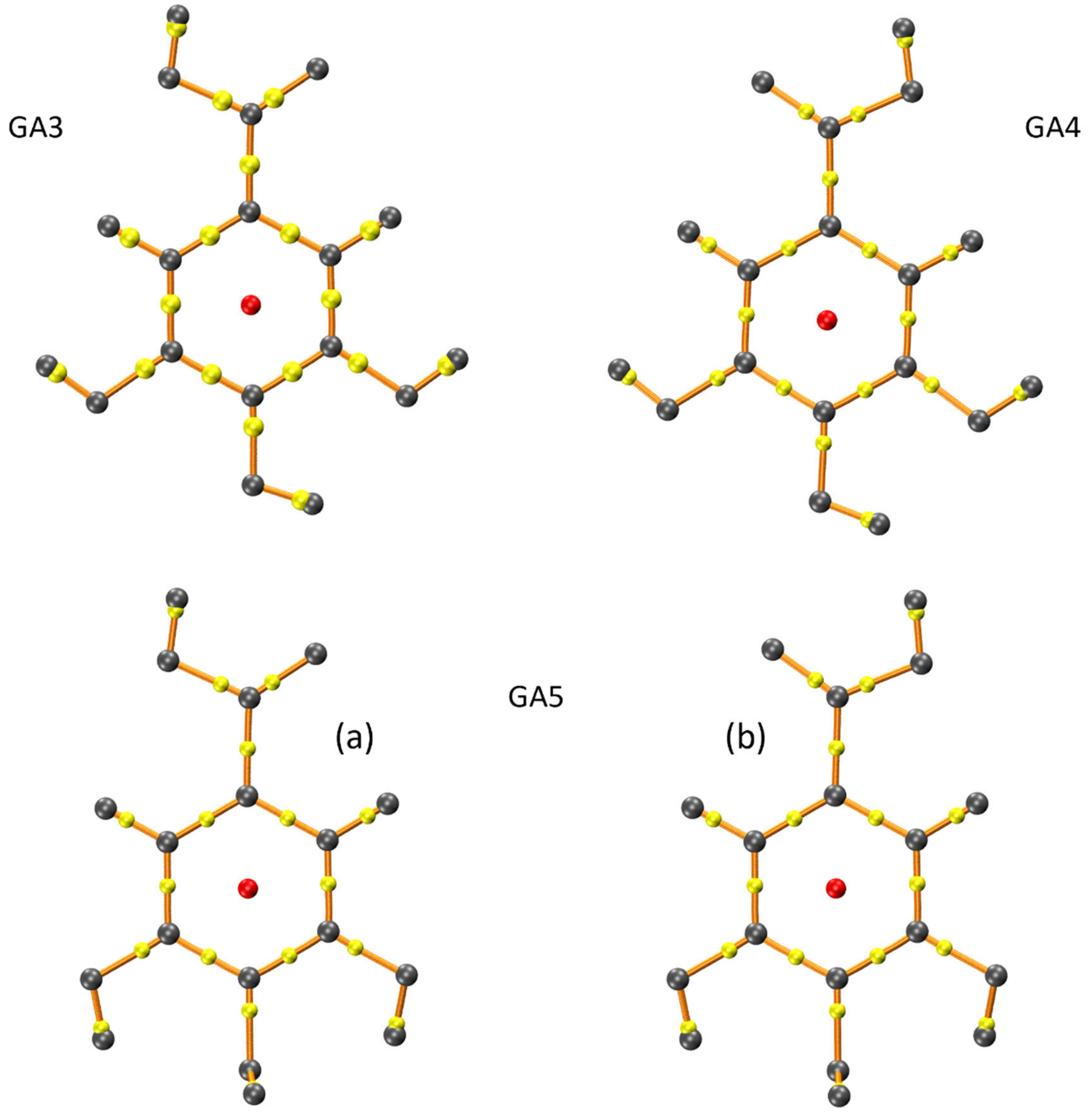

Table S1. Rotational parameters, electric dipole moment components, and energies for the five conformers of gallic acid calculated at B3LYP-D3/6-311++G(2d,p) level of theory.

Param.[a]	**GA1**	**GA2**	**GA3**	**GA4**	**GA5**
A/MHz	1602.4	1602.3	1596.4	1595.8	1600.4
B/MHz	663.6	663.3	663.8	663.8	662.3
C/MHz	469.3	469.1	468.9	468.8	469.2
κ	-0.66	-0.66	-0.65	-0.65	-0.66
P_a/uÅ^2	761.5	761.9	761.3	761.3	762.2
P_b/uÅ^2	315.3	315.4	316.5	316.7	314.9
P_c/uÅ^2	0.0	0.0	0.0	0.0	0.9
μ_a/D	1.9	1.8	0.3	0.3	3.3
μ_b/D	1.4	4.2	0.0	2.8	1.4
μ_c/D	0.0	0.0	0.0	0.0	0.9
ΔE /cm^{-1}	0.0	160	1265	1288	1280
ΔG /cm^{-1}	0.0	116	1162	1161	1355

[a] A, B and C are the rotational constants. κ is the Ray's asymmetry parameter ($-1<\kappa=(2B-A-C)/(A-C)<1$). P_α ($\alpha= a$, b or c), are the planar moments of inertia derived from the inertial moments I_α (*i.e.* $P_c=(I_a+I_b-I_c)/2$). μ_a, μ_b, μ_c, are the electric dipole moment components along the principal inertial axes. ΔE and ΔG are the relative electronic and Gibbs energy related to GA1 conformer that has values of -646.7245244 Hartree and -646.633306 Hartree respectively. (1 kJ/mol = 83.5935 cm^{-1}).

Table S2. Rotational parameters, electric dipole moment components, and energies for the five conformers of gallic acid calculated at B3LYP-D3BJ/6-311++G(2d,p) level of theory.

Param.[a]	**GA1**	**GA2**	**GA3**	**GA4**	**GA5**
A/MHz	1607.1	1607.0	1598.8	1598.1	1605.8
B/MHz	664.6	664.2	665.1	665.1	663.0
C/MHz	470.2	470.0	469.7	469.7	470.0
κ	-0.66	-0.66	-0.65	-0.65	-0.66
P_a/uÅ^2	760.4	760.8	759.9	759.8	761.4
P_b/uÅ^2	314.4	314.4	316.1	316.2	313.8
P_c/uÅ^2	0.0	0.0	0.0	0.0	0.9
μ_a/D	1.9	1.8	0.3	0.3	3.3
μ_b/D	1.4	4.2	0.0	2.8	1.4
μ_c/D	0.0	0.0	0.0	0.0	0.9
ΔE /cm^{-1}	0.0	158	1237	1260	1367
ΔG /cm^{-1}	0.0	120	1139	1144	1416

[a] A, B and C are the rotational constants. κ is the Ray's asymmetry parameter ($-1<\kappa=(2B-A-C)/(A-C)<1$). P_α ($\alpha= a$, b or c), are the planar moments of inertia derived from the inertial moments I_α (*i.e.* $P_c=(I_a+I_b-I_c)/2$). μ_a, μ_b, μ_c, are the electric dipole moment components along the principal inertial axes. ΔE and ΔG are the relative electronic and Gibbs energy related to GA1 conformer that has values of -646.7435645 Hartree and -646.652142 Hartree respectively. (1 kJ/mol = 83.5935 cm^{-1}).

Table S3. Rotational parameters, electric dipole moment components, and energies for the five conformers of gallic acid calculated at B3LYP-D3BJ/def2TZVP level of theory.

Param.[a]	**GA1**	**GA2**	**GA3**	**GA4**	**GA5**
A/MHz	1609.4	1609.3	1600.5	1599.8	1608.5
B/MHz	664.9	664.6	665.5	665.6	663.4
C/MHz	470.5	470.3	470.1	470.0	470.5
κ	-0.66	-0.66	-0.65	-0.65	-0.66
P_a/uÅ^2	760.1	760.5	759.3	759.3	760.9
P_b/uÅ^2	314.0	314.1	315.7	315.9	313.3
P_c/uÅ^2	0.0	0.0	0.0	0.0	0.9
μ_a/D	1.9	1.9	0.2	0.3	3.4
μ_b/D	1.4	4.2	0.0	2.8	1.4
μ_c/D	0.0	0.0	0.0	0.0	1.0
ΔE /cm^{-1}	0.0	159	1200	1223	1421
ΔG /cm^{-1}	0.0	131	1158	1172	1379

[a] A, B and C are the rotational constants. κ is the Ray's asymmetry parameter ($-1<\kappa=(2B-A-C)/(A-C)<1$). P_α ($\alpha = a$, b or c), are the planar moments of inertia derived from the inertial moments I_α (*i.e.* $P_c=(I_a+I_b-I_c)/2$). μ_a, μ_b, μ_c, are the electric dipole moment components along the principal inertial axes. ΔE and ΔG are the relative electronic and Gibbs energy related to GA1 conformer that has values of -646.7979253 Hartree and -646.706264 Hartree respectively. (1 kJ/mol = 83.5935 cm^{-1}).

Table S4. Rotational parameters, electric dipole moment components, and energies for the five conformers of gallic acid calculated at MP2/6-311++G(2d,p) level of theory.

Param.[a]	**GA1**	**GA2**	**GA3**	**GA4**	**GA5**
A/MHz	1602.1	1602.0	1596.0	1595.4	1596.9
B/MHz	663.4	663.0	663.2	663.3	662.4
C/MHz	469.1	468.9	468.5	468.5	469.0
κ	-0.66	-0.66	-0.65	-0.65	-0.66
P_a/uÅ^2	761.8	762.3	762.0	761.9	762.0
P_b/uÅ^2	315.5	315.5	316.7	316.8	315.5
P_c/uÅ^2	0.0	0.0	0.0	0.0	0.9
μ_a/D	1.6	1.3	0.6	0.6	3.2
μ_b/D	1.4	4.2	0.3	2.7	1.3
μ_c/D	0.8	0.7	0.2	0.4	0.7
ΔE /cm^{-1}	0.0	136	1270	1286	1307
ΔG /cm^{-1}	0.0	113	1091	1150	1604

[a] A, B and C are the rotational constants. κ is the Ray's asymmetry parameter ($-1<\kappa=(2B-A-C)/(A-C)<1$). P_α ($\alpha = a$, b or c), are the planar moments of inertia derived from the inertial moments I_α (*i.e.* $P_c=(I_a+I_b-I_c)/2$). μ_a, μ_b, μ_c, are the electric dipole moment components along the principal inertial axes. ΔE and ΔG are the relative electronic and Gibbs energy related to GA1 conformer that has values of -645.1706947 Hartree and -645.080852 Hartree respectively. (1 kJ/mol = 83.5935 cm^{-1}).

Table S5. Rotational parameters, electric dipole moment components, and energies for the five conformers of gallic acid calculated at MP2/aug-cc-PVDZ level of theory.

Param.[a]	**GA1**	**GA2**	**GA3**	**GA4**	**GA5**
A/MHz	1579.5	1579.4	1575.5	1574.9	1572.3
B/MHz	654.8	654.4	654.3	654.3	654.2
C/MHz	462.9	462.7	462.3	462.3	462.7
κ	-0.66	-0.66	-0.66	-0.65	-0.65
P_a/uÅ^2	771.8	772.3	772.4	772.3	771.6
P_b/uÅ^2	320.0	320.0	320.8	320.8	320.5
P_c/uÅ^2	0.0	0.0	0.0	0.0	0.9
μ_a/D	1.5	1.3	0.5	0.5	3.2
μ_b/D	1.4	4.1	0.3	2.6	1.3
μ_c/D	0.8	0.8	0.1	0.3	1.0
ΔE /cm^{-1}	0.0	138	1163	1179	1334

[a] A, B and C are the rotational constants. κ is the Ray's asymmetry parameter ($-1<\kappa=(2B-A-C)/(A-C)<1$). P_α ($\alpha= a$, b or c), are the planar moments of inertia derived from the inertial moments I_α (*i.e.* $P_c=(I_a+I_b-I_c)/2$). μ_a, μ_b, μ_c, are the electric dipole moment components along the principal inertial axes. ΔE is the relative electronic energy related to GA1 conformer that has a value of -644.9228737. (1 kJ/mol = 83.5935 cm^{-1}).

Table S6. Rotational parameters, electric dipole moment components, and energies for the five conformers of gallic acid calculated at MP2/aug-cc-PVTZ level of theory.

Param.[a]	**GA1**	**GA2**	**GA3**	**GA4**	**GA5**
A/MHz	1607.2	1607.1	1601.1	1600.2	1601.6
B/MHz	665.6	665.2	665.2	665.5	664.6
C/MHz	470.7	470.5	469.9	470.0	470.5
κ	-0.66	-0.66	-0.65	-0.65	-0.66
P_a/uÅ^2	759.3	759.7	759.8	759.4	759.5
P_b/uÅ^2	314.4	314.4	315.7	315.8	314.6
P_c/uÅ^2	0.0	0.0	0.0	0.0	0.9
μ_a/D	1.5	1.7	0.5	0.5	3.3
μ_b/D	1.6	4.0	0.1	2.6	0.6
μ_c/D	0.0	0.0	0.0	0	1.3
ΔE /cm^{-1}	0.0	142	1186	1206	1464

[a] A, B and C are the rotational constants. κ is the Ray's asymmetry parameter ($-1<\kappa=(2B-A-C)/(A-C)<1$). P_α ($\alpha= a$, b or c), are the planar moments of inertia derived from the inertial moments I_α (*i.e.* $P_c=(I_a+I_b-I_c)/2$). μ_a, μ_b, μ_c, are the electric dipole moment components along the principal inertial axes. ΔE is the relative electronic energy related to GA1 conformer that has a value of -645.4631638 Hartree. (1 kJ/mol = 83.5935 cm^{-1}).

Table S7: Predicted coordinates of the molecular structure of GA1 conformer calculated at B3LYP-GD3/6-311++G(2d,p) level of theory.

Atom	*a*	*b*	*c*
**C-1**	1.050	-0.025	0.000
**C-2**	0.376	1.196	0.000
**C-3**	-1.011	1.214	0.000
**C-4**	-1.723	0.014	0.000
**C-5**	-1.040	-1.203	0.000
**C-6**	0.342	-1.230	0.000
**C-7**	2.529	-0.105	0.000
**O-8**	3.166	-1.134	0.000
**O-9**	3.132	1.111	0.000
**O-10**	-1.667	2.408	0.000
**O-11**	-3.087	0.086	0.000
**O-12**	-1.844	-2.319	0.000
**H-13**	0.915	2.132	0.000
**H-14**	0.887	-2.166	0.000
**H-15**	4.087	0.943	0.000
**H-16**	-2.620	2.243	0.000
**H-17**	-3.451	-0.810	0.000
**H-18**	-1.306	-3.117	0.000

Table S8: Predicted coordinates of the molecular structure of GA2 conformer calculated at B3LYP-GD3/6-311++G(2d,p) level of theory.

Atom	*a*	*b*	*c*
**C-1**	1.050	-0.029	0.000
**C-2**	0.378	1.198	0.000
**C-3**	-1.006	1.207	0.000
**C-4**	-1.723	0.011	0.000
**C-5**	-1.045	-1.210	0.000
**C-6**	0.340	-1.228	0.000
**C-7**	2.529	-0.101	0.000
**O-8**	3.176	-1.122	0.000
**O-9**	3.125	1.123	0.000
**O-10**	-1.779	2.346	0.000
**O-11**	-3.089	-0.021	0.000
**O-12**	-1.735	-2.383	0.000
**H-13**	0.933	2.127	0.000
**H-14**	0.867	-2.172	0.000
**H-15**	4.080	0.959	0.000
**H-16**	-1.220	3.130	0.000
**H-17**	-3.427	0.884	0.000
**H-18**	-2.683	-2.192	0.000

Table S9: Predicted coordinates of the molecular structure of GA3 conformer calculated at B3LYP-GD3/6-311++G(2d,p) level of theory.

Atom	*a*	*b*	*c*
**C-1**	1.053	-0.033	0.000
**C-2**	0.376	1.188	0.000
**C-3**	-1.012	1.220	0.000
**C-4**	-1.738	0.024	0.000
**C-5**	-1.045	-1.192	0.000
**C-6**	0.337	-1.230	0.000
**C-7**	2.528	-0.113	0.000
**O-8**	3.168	-1.141	0.000
**O-9**	3.133	1.105	0.000
**O-10**	-1.737	2.376	0.000
**O-11**	-3.092	0.067	0.000
**O-12**	-1.848	-2.308	0.000
**H-13**	0.935	2.115	0.000
**H-14**	0.874	-2.171	0.000
**H-15**	4.087	0.935	0.000
**H-16**	-1.137	3.130	0.000
**H-17**	-3.426	-0.841	0.000
**H-18**	-1.310	-3.107	0.000

Table S10: Predicted coordinates of the molecular structure of GA4 conformer calculated at B3LYP-GD3/6-311++G(2d,p) level of theory.

Atom	*a*	*b*	*c*
**C-1**	1.053	0.020	0.000
**C-2**	0.341	1.219	0.000
**C-3**	-1.046	1.215	0.000
**C-4**	-1.738	-0.002	0.000
**C-5**	-1.012	-1.197	0.000
**C-6**	0.372	-1.199	0.000
**C-7**	2.529	0.092	0.000
**O-8**	3.175	1.116	0.000
**O-9**	3.126	-1.130	0.000
**O-10**	-1.801	2.351	0.000
**O-11**	-3.093	0.004	0.000
**O-12**	-1.784	-2.335	0.000
**H-13**	0.890	2.153	0.000
**H-14**	0.920	-2.133	0.000
**H-15**	4.081	-0.965	0.000
**H-16**	-1.221	3.120	0.000
**H-17**	-3.402	-0.912	0.000
**H-18**	-1.226	-3.119	0.000

Table S11: Predicted coordinates of the molecular structure of GA5 conformer calculated at B3LYP-GD3/6-311++G(2d,p) level of theory.

Atom	*a*	*b*	*c*
**C-1**	1.044	-0.027	-0.001
**C-2**	0.380	1.200	0.008
**C-3**	-1.009	1.215	0.007
**C-4**	-1.714	0.013	-0.022
**C-5**	-1.043	-1.211	0.008
**C-6**	0.343	-1.233	0.007
**C-7**	2.529	-0.103	-0.006
**O-8**	3.168	-1.128	-0.010
**O-9**	3.126	1.117	-0.006
**O-10**	-1.667	2.407	0.019
**O-11**	-3.109	0.034	0.008
**O-12**	-1.736	-2.382	0.020
**H-13**	0.921	2.134	0.021
**H-14**	0.874	-2.175	0.019
**H-15**	4.081	0.954	-0.009
**H-16**	-2.605	2.236	0.181
**H-17**	-3.454	0.032	-0.895
**H-18**	-2.669	-2.184	0.186

Table S12: Observed rotational transitions and residuals (all the values in MHz), fitted rotational parameters with standard errors, and correlation coefficients of the fit for R1 rotamer.

	J´	K´$_{-1}$	K´$_{+1}$	J”	K”$_{-1}$	K”$_{+1}$	Obs.	Obs.-Cal.	Error
1:	2	0	2	1	0	1	2238.8424	0.0049	0.015
2:	4	1	3	4	0	4	2302.0875	-0.0042	0.015
3:	2	1	1	1	1	0	2459.8921	0.0050	0.015
4:	3	0	3	2	1	2	2521.4940	0.0048	0.015
5:	4	2	2	4	1	3	2573.7596	-0.0034	0.015
6:	3	2	1	3	1	2	2677.3715	0.0011	0.015
7:	6	2	4	6	1	5	2818.3292	-0.0041	0.015
8:	2	1	2	1	0	1	3011.2778	-0.0006	0.015
9:	5	1	4	5	0	5	3074.2266	0.0056	0.015
10:	3	1	3	2	1	2	3091.9814	0.0040	0.015
11:	7	2	5	7	1	6	3255.8844	0.0006	0.015
12:	3	0	3	2	0	2	3293.9287	-0.0013	0.015
13:	3	2	2	2	2	1	3398.8409	-0.0008	0.015
14:	2	2	1	2	1	2	3400.1133	0.0105	0.015
15:	3	2	1	2	2	0	3503.7542	0.0005	0.015
16:	3	1	2	2	1	1	3671.5668	-0.0002	0.015
17:	3	2	2	3	1	3	3706.9645	-0.0026	0.015
18:	4	0	4	3	1	3	3717.4233	-0.0016	0.015
19:	5	1	4	4	2	3	3807.2779	0.0040	0.015
20:	3	1	3	2	0	2	3864.4191	0.0008	0.015
21:	8	3	5	8	2	6	3911.1341	-0.0016	0.015
22:	8	2	6	8	1	7	3932.5759	-0.0013	0.015
23:	9	3	6	9	2	7	3933.9765	-0.0038	0.015
24:	6	1	5	6	0	6	4016.3984	-0.0022	0.015
25:	7	3	4	7	2	5	4076.2957	0.0030	0.015
26:	4	1	4	3	1	3	4096.1143	-0.0010	0.015
27:	4	2	3	4	1	4	4121.4974	0.0036	0.015
28:	10	3	7	10	2	8	4197.7014	-0.0002	0.015
29:	4	0	4	3	0	3	4287.9136	0.0004	0.015
30:	6	3	3	6	2	4	4353.7968	-0.0033	0.015
31:	7	2	5	6	3	4	4393.9923	0.0026	0.015
32:	9	3	6	8	4	5	4501.4082	0.0095	0.015
33:	4	2	3	3	2	2	4510.6414	-0.0006	0.015
34:	4	3	2	3	3	1	4579.4739	-0.0027	0.015
35:	4	3	1	3	3	0	4593.6095	0.0000	0.015
36:	5	2	4	5	1	5	4642.6986	-0.0025	0.015
37:	5	3	2	5	2	3	4656.0790	0.0018	0.015
38:	4	1	4	3	0	3	4666.6032	-0.0003	0.015
39:	11	3	8	11	2	9	4731.1691	0.0026	0.015
40:	4	2	2	3	2	1	4754.3427	-0.0004	0.015
41:	9	2	7	9	1	8	4831.2034	-0.0011	0.015
42:	5	0	5	4	1	4	4854.5458	-0.0009	0.015
43:	4	1	3	3	1	2	4857.9498	-0.0006	0.015
44:	4	3	1	4	2	2	4907.4168	0.0015	0.015
45:	7	1	6	7	1	7	4995.9495	0.0046	0.015
46:	7	1	6	7	0	7	5064.6774	-0.0008	0.015
47:	3	3	0	3	2	1	5068.1468	-0.0020	0.015
48:	5	1	5	4	1	4	5083.4996	-0.0006	0.015
49:	12	4	8	12	3	9	5150.4286	-0.0090	0.015
50:	3	3	1	3	2	2	5197.7329	0.0011	0.015
51:	5	0	5	4	0	4	5233.2371	0.0000	0.015
52:	6	2	5	6	1	6	5264.5359	0.0008	0.015
53:	4	3	2	4	2	3	5266.5652	-0.0011	0.015

54:	2	2	1	1	1	0	5278.0132	-0.0001	0.015
55:	6	1	5	5	2	4	5300.6515	0.0007	0.015
56:	11	4	7	11	3	8	5335.3212	-0.0007	0.015
57:	5	3	3	5	2	4	5397.2606	-0.0004	0.015
58:	5	1	5	4	0	4	5462.1904	-0.0002	0.015
59:	2	2	0	1	1	1	5499.0644	0.0014	0.015
60:	12	3	9	12	2	10	5533.2827	0.0000	0.015
61:	14	4	10	14	3	11	5552.1507	0.0074	0.015
62:	5	2	4	4	2	3	5604.7069	-0.0008	0.015
63:	6	3	4	6	2	5	5609.1619	0.0017	0.015
64:	10	2	8	10	2	9	5664.3209	-0.0069	0.015
65:	10	4	6	10	3	7	5685.5537	-0.0011	0.015
66:	5	4	2	4	4	1	5724.7554	-0.0026	0.015
67:	5	4	1	4	4	0	5726.1384	-0.0022	0.015
68:	5	3	3	4	3	2	5735.3999	-0.0025	0.015
69:	5	3	2	4	3	1	5783.4836	-0.0003	0.015
70:	10	2	8	10	1	9	5895.7435	-0.0004	0.015
71:	7	3	5	7	2	6	5917.4619	-0.0027	0.015
72:	6	0	6	5	1	5	5926.9515	0.0002	0.015
73:	7	2	6	7	1	7	5975.5800	0.0006	0.015
74:	5	1	4	4	1	3	6005.3651	-0.0012	0.015
75:	5	2	3	4	2	2	6034.8215	-0.0006	0.015
76:	7	2	6	7	0	7	6044.3077	-0.0049	0.015
77:	6	1	6	5	1	5	6055.7181	-0.0010	0.015
78:	9	4	5	9	3	6	6105.7476	0.0003	0.015
79:	8	2	6	7	3	5	6121.1606	-0.0043	0.015
80:	8	1	7	8	0	8	6149.5828	-0.0014	0.015
81:	6	0	6	5	0	5	6155.9043	-0.0005	0.015
82:	3	2	2	2	1	1	6216.9681	0.0002	0.015
83:	6	1	6	5	0	5	6284.6729	0.0001	0.015
84:	8	3	6	8	2	7	6331.3263	-0.0003	0.015
85:	8	4	4	8	3	5	6503.3142	0.0000	0.015
86:	6	2	5	5	2	4	6677.5518	-0.0012	0.015
87:	7	1	6	6	2	5	6747.2643	-0.0017	0.015
88:	8	2	7	8	1	8	6760.3745	-0.0020	0.015
89:	7	4	3	7	3	4	6815.0633	-0.0008	0.015
90:	9	3	7	9	2	8	6852.7873	0.0048	0.015
91:	6	5	2	5	5	1	6865.8833	-0.0019	0.015
92:	6	5	1	5	5	0	6866.0024	0.0028	0.015
93:	6	4	3	5	4	2	6887.7775	0.0000	0.015
94:	6	3	4	5	3	3	6889.4511	-0.0008	0.015
95:	6	4	2	5	4	1	6893.9026	-0.0020	0.015
96:	11	2	9	11	2	10	6918.5219	-0.0012	0.015
97:	3	2	1	2	1	2	6930.9140	0.0000	0.015
98:	7	0	7	6	1	6	6947.1242	0.0014	0.015
99:	6	3	3	5	3	2	7010.9217	-0.0004	0.015
100:	7	1	7	6	1	6	7015.8570	0.0007	0.015
101:	6	4	2	6	3	3	7021.4686	0.0009	0.015
102:	11	2	9	11	1	10	7047.3799	0.0003	0.015
103:	4	2	3	3	1	2	7056.0446	0.0017	0.015
104:	7	0	7	6	0	6	7075.8909	0.0003	0.015
105:	6	1	5	5	1	4	7098.0840	-0.0005	0.015
106:	5	4	1	5	3	2	7138.4863	0.0011	0.015
107:	7	1	7	6	0	6	7144.6243	0.0003	0.015
108:	4	4	0	4	3	1	7195.8314	0.0029	0.015
109:	6	4	3	6	3	4	7199.8529	-0.0019	0.015
110:	5	4	2	5	3	3	7201.5290	-0.0003	0.015
111:	4	4	1	4	3	2	7212.1720	-0.0017	0.015

112:	9	1	8	9	0	9	7221.6990	-0.0018	0.015
113:	7	4	4	7	3	5	7222.5536	0.0012	0.015
114:	8	4	5	8	3	6	7289.4529	0.0013	0.015
115:	6	2	4	5	2	3	7313.1990	0.0000	0.015
116:	9	4	6	9	3	7	7421.9821	-0.0037	0.015
117:	10	3	8	10	2	9	7476.6519	0.0073	0.015
118:	9	2	8	9	1	9	7601.8093	0.0030	0.015
119:	7	2	6	6	2	5	7726.8990	-0.0015	0.015
120:	5	2	4	4	1	3	7802.7981	-0.0020	0.015
121:	9	2	7	8	3	6	7856.8772	0.0073	0.015
122:	8	0	8	7	1	7	7932.0493	0.0019	0.015
123:	8	1	8	7	1	7	7967.3856	0.0012	0.015
124:	4	2	3	4	0	4	4500.1735	-0.0106	0.015
125:	5	2	4	5	0	5	4871.6577	0.0029	0.015
126:	6	2	5	6	0	6	5393.3052	0.0023	0.015
127:	10	3	7	9	4	6	6324.9848	-0.0100	0.015
128:	13	3	10	13	2	11	6563.7119	-0.0020	0.015
129:	8	2	7	8	0	8	6795.7186	0.0050	0.015
130:	7	3	5	7	1	6	6897.1016	0.0024	0.015
131:	6	3	4	6	1	5	6986.0652	0.0028	0.015
132:	9	3	7	9	1	8	7250.5579	-0.0008	0.015
133:	13	5	8	13	4	9	7403.8871	0.0015	0.015

PARAMETERS IN FIT WITH STANDARD ERRORS ON THOSE THAT ARE FITTED:

10000	A	/MHz	1602.84558(20)
20000	B	/MHz	663.46993(10)
30000	C	/MHz	469.477690(86)
200	D_J	/MHz	0.00000690(86)
1100	D_{JK}	/MHz	0.0000190(20)
2000	D_K	/MHz	0.000037(11)
40100	d_J	/MHz	0.00000209(23)
50000	d_k	/MHz	0.000000510(88)

CORRELATION COEFFICIENTS, C_{ij}:

1.0000							
0.2513	1.0000						
0.2582	0.5486	1.0000					
-0.1857	-0.8305	-0.7219	1.0000				
-0.1492	-0.2829	-0.0338	0.1282	1.0000			
-0.6306	0.2093	0.0594	-0.1389	-0.5443	1.0000		
-0.0762	-0.5555	0.3391	0.2104	0.2892	-0.1317	1.0000	
0.1368	0.3847	-0.2128	-0.1837	-0.1687	0.0089	-0.7615	1.0000

Table S13: Observed rotational transitions and residuals (all the values in MHz), fitted rotational parameters with standard errors, and correlation coefficients of the fit for R2 rotamer.

	J´	K´-1	K´+1	J"	K"-1	K"+1	Obs.	Obs.-Cal.	Error
1:	4	1	3	4	0	4	2300.9233	0.0073	0.015
2:	4	2	2	4	1	3	2574.1653	-0.0004	0.015
3:	5	2	3	5	1	4	2603.2340	-0.0057	0.015
4:	3	2	1	3	1	2	2677.9094	-0.0062	0.015
5:	6	2	4	6	1	5	2817.7070	0.0034	0.015
6:	2	2	0	2	1	1	2845.6971	-0.0011	0.015
7:	2	1	2	1	0	1	3010.6151	-0.0019	0.015
8:	5	1	4	5	0	5	3072.2927	-0.0029	0.015
9:	3	1	3	2	1	2	3090.7407	0.0092	0.015
10:	7	2	5	7	1	6	3254.3297	0.0004	0.015
11:	3	0	3	2	0	2	3292.5928	0.0005	0.015
12:	3	1	2	2	1	1	3669.8407	0.0020	0.015
13:	3	2	2	3	1	3	3706.7697	0.0010	0.015
14:	4	0	4	3	1	3	3715.3969	0.0000	0.015
15:	5	1	4	4	2	3	3803.5990	0.0019	0.015
16:	3	1	3	2	0	2	3863.4752	0.0002	0.015
17:	8	3	5	8	2	6	3911.9240	-0.0001	0.015
18:	8	2	6	8	1	7	3929.8843	-0.0036	0.015
19:	6	1	5	6	0	6	4013.7039	-0.0064	0.015
20:	7	3	4	7	2	5	4077.5792	0.0001	0.015
21:	4	1	4	3	1	3	4094.4967	-0.0002	0.015
22:	4	2	3	4	1	4	4120.9231	-0.0044	0.015
23:	4	0	4	3	0	3	4286.2719	-0.0076	0.015
24:	11	3	9	10	4	6	4345.7539	-0.0026	0.015
25:	6	3	3	6	2	4	4355.2003	0.0014	0.015
26:	7	2	5	6	3	4	4387.9974	-0.0009	0.015
27:	9	3	6	8	4	5	4493.3570	0.0100	0.015
28:	4	2	3	3	2	2	4508.6555	-0.0004	0.015
29:	4	3	1	3	3	0	4591.4678	-0.0005	0.015
30:	5	2	4	5	1	5	4641.6828	0.0023	0.015
31:	12	4	9	11	5	6	4654.7664	0.0024	0.015
32:	5	3	2	5	2	3	4657.3127	0.0022	0.015
33:	4	1	4	3	0	3	4665.3795	-0.0001	0.015
34:	4	2	2	3	2	1	4751.9664	-0.0015	0.015
35:	9	2	7	9	1	8	4827.3468	0.0087	0.015
36:	5	0	5	4	1	4	4852.2278	-0.0011	0.015
37:	4	1	3	3	1	2	4855.7187	0.0007	0.015
38:	4	3	1	4	2	2	4908.3726	-0.0002	0.015
39:	7	1	6	7	0	7	5061.3471	0.0020	0.015
40:	3	3	0	3	2	1	5068.8703	-0.0020	0.015
41:	5	1	5	4	1	4	5081.5382	0.0070	0.015
42:	3	3	1	3	2	2	5198.2318	-0.0023	0.015
43:	5	0	5	4	0	4	5231.3290	-0.0001	0.015
44:	6	2	5	6	1	6	5262.9923	-0.0014	0.015
45:	4	3	2	4	2	3	5266.9496	-0.0021	0.015
46:	2	2	1	1	1	0	5277.4003	-0.0005	0.015
47:	6	1	5	5	2	4	5296.4214	0.0000	0.015
48:	11	4	7	11	3	8	5337.4375	-0.0051	0.015
49:	5	3	3	5	2	4	5397.4370	0.0035	0.015
50:	5	1	5	4	0	4	5460.6339	0.0025	0.015
51:	2	2	0	1	1	1	5498.2415	0.0017	0.015
52:	5	2	4	4	2	3	5602.2840	-0.0001	0.015
53:	6	3	4	6	2	5	5609.0068	0.0030	0.015

54:	10	4	6	10	3	7	5688.1421	0.0002	0.015
55:	5	4	2	4	4	1	5722.1281	0.0065	0.015
56:	5	4	1	4	4	0	5723.4979	-0.0011	0.015
57:	5	3	3	4	3	2	5732.7654	-0.0003	0.015
58:	5	3	2	4	3	1	5780.7199	-0.0006	0.015
59:	10	2	8	10	1	9	5890.8707	0.0003	0.015
60:	7	3	5	7	2	6	5916.8720	0.0141	0.015
61:	6	0	6	5	1	5	5924.3929	0.0012	0.015
62:	7	2	6	7	1	7	5973.4771	-0.0028	0.015
63:	5	1	4	4	1	3	6002.7083	-0.0005	0.015
64:	5	2	3	4	2	2	6031.7814	-0.0014	0.015
65:	6	1	6	5	1	5	6053.4144	0.0002	0.015
66:	9	4	5	9	3	6	6108.2689	0.0008	0.015
67:	8	2	6	7	3	5	6114.3206	-0.0036	0.015
68:	8	1	7	8	0	8	6145.7917	0.0010	0.015
69:	6	0	6	5	0	5	6153.6952	0.0014	0.015
70:	3	2	2	2	1	1	6216.0126	0.0002	0.015
71:	6	1	6	5	0	5	6282.7155	-0.0008	0.015
72:	10	3	7	9	4	6	6315.6390	-0.0026	0.015
73:	8	4	4	8	3	5	6505.4348	0.0029	0.015
74:	6	2	5	5	2	4	6674.7269	-0.0004	0.015
75:	7	1	6	6	2	5	6742.6731	0.0001	0.015
76:	8	2	7	8	1	8	6757.7132	0.0013	0.015
77:	7	4	3	7	3	4	6816.6901	0.0025	0.015
78:	9	3	7	9	2	8	6850.9597	-0.0016	0.015
79:	6	5	2	5	5	1	6862.7148	-0.0114	0.015
80:	6	5	1	5	5	0	6862.8380	-0.0020	0.015
81:	6	4	3	5	4	2	6884.5815	-0.0036	0.015
82:	6	3	4	5	3	3	6886.3003	0.0027	0.015
83:	6	4	2	5	4	1	6890.6919	0.0017	0.015
84:	3	2	1	2	1	2	6929.2409	-0.0010	0.015
85:	7	0	7	6	1	6	6944.3215	-0.0001	0.015
86:	6	3	3	5	3	2	7007.4612	0.0005	0.015
87:	7	1	7	6	1	6	7013.2227	0.0006	0.015
88:	6	4	2	6	3	3	7022.6901	0.0001	0.015
89:	11	2	9	11	1	10	7041.8182	0.0050	0.015
90:	4	2	3	3	1	2	7054.8299	0.0001	0.015
91:	7	0	7	6	0	6	7073.3440	0.0000	0.015
92:	6	1	5	5	1	4	7095.1078	-0.0007	0.015
93:	5	4	1	5	3	2	7139.4610	0.0005	0.015
94:	7	1	7	6	0	6	7142.2449	0.0002	0.015
95:	4	4	0	4	3	1	7196.6811	-0.0008	0.015
96:	9	1	8	9	1	9	7199.8529	-0.0076	0.015
97:	6	4	3	6	3	4	7200.6249	-0.0015	0.015
98:	5	4	2	5	3	3	7202.3384	-0.0005	0.015
99:	4	4	1	4	3	2	7212.9808	-0.0023	0.015
100:	9	1	8	9	0	9	7217.5926	0.0026	0.015
101:	7	4	4	7	3	5	7223.2120	-0.0001	0.015
102:	8	4	5	8	3	6	7289.8849	-0.0040	0.015
103:	6	2	4	5	2	3	7309.5711	-0.0012	0.015
104:	13	5	8	13	4	9	7408.0206	0.0019	0.015
105:	9	4	6	9	3	7	7422.0659	0.0045	0.015
106:	10	3	8	10	2	9	7474.1158	-0.0032	0.015
107:	9	2	8	9	1	9	7598.5957	0.0024	0.015
108:	10	4	7	10	3	8	7639.4857	0.0040	0.015
109:	7	2	6	6	2	5	7723.7126	0.0043	0.015
110:	5	2	4	4	1	3	7801.3950	-0.0009	0.015
111:	9	2	7	8	3	6	7849.3703	-0.0054	0.015

112:	8	0	8	7	1	7	7928.9830	-0.0003	0.015
113:	12	5	7	12	4	8	7931.4308	0.0037	0.015
114:	11	4	8	11	3	9	7957.3281	-0.0118	0.015
115:	8	1	8	7	1	7	7964.4213	-0.0008	0.015
116:	8	0	8	7	0	7	7997.8861	0.0021	0.015

PARAMETERS IN FIT WITH STANDARD ERRORS ON THOSE THAT ARE FITTED:

10000	A	/MHz	1602.69867(25)
20000	B	/MHz	663.13549(10)
30000	C	/MHz	469.30622(10)
200	D_J	/MHz	0.00000605(63)
1100	D_{JK}	/MHz	0.0000274(26)
2000	D_K	/MHz	0.000039(13)
40100	d_J	/MHz	0.00000253(33)
50000	d_k	/MHz	0.00000025(11)

CORRELATION COEFFICIENTS, C_{ij}:

1.0000						
0.3506	1.0000					
0.1471	0.2583	1.0000				
-0.2467	-0.6517	-0.6833	1.0000			
-0.0148	-0.2819	0.0455	0.0318	1.0000		
-0.6786	0.0143	0.0293	0.0836	-0.6361	1.0000	
-0.2074	-0.5737	0.5866	-0.0347	0.2646	0.0620	1.0000
0.1483	-0.0182	-0.3942	0.3139	0.1891	-0.2630	-0.3781
